# Identification of Genome-Wide Mutations in Ciprofloxacin-Resistant *F*. *tularensis* LVS Using Whole Genome Tiling Arrays and Next Generation Sequencing

**DOI:** 10.1371/journal.pone.0163458

**Published:** 2016-09-26

**Authors:** Crystal J. Jaing, Kevin S. McLoughlin, James B. Thissen, Adam Zemla, Shea N. Gardner, Lisa M. Vergez, Feliza Bourguet, Shalini Mabery, Viacheslav Y. Fofanov, Heather Koshinsky, Paul J. Jackson

**Affiliations:** 1 Physical Life Sciences Directorate, Lawrence Livermore National Laboratory, Livermore, CA, 94550, United States of America; 2 Computations Directorate, Lawrence Livermore National Laboratory, Livermore, CA, 94550, United States of America; 3 Eureka Genomics, Hercules, CA, 94547, United States of America; Mayo Clinic Arizona, UNITED STATES

## Abstract

*Francisella tularensis* is classified as a Class A bioterrorism agent by the U.S. government due to its high virulence and the ease with which it can be spread as an aerosol. It is a facultative intracellular pathogen and the causative agent of tularemia. Ciprofloxacin (Cipro) is a broad spectrum antibiotic effective against Gram-positive and Gram-negative bacteria. Increased Cipro resistance in pathogenic microbes is of serious concern when considering options for medical treatment of bacterial infections. Identification of genes and loci that are associated with Ciprofloxacin resistance will help advance the understanding of resistance mechanisms and may, in the future, provide better treatment options for patients. It may also provide information for development of assays that can rapidly identify Cipro-resistant isolates of this pathogen. In this study, we selected a large number of *F*. *tularensis* live vaccine strain (LVS) isolates that survived in progressively higher Ciprofloxacin concentrations, screened the isolates using a whole genome *F*. *tularensis* LVS tiling microarray and Illumina sequencing, and identified both known and novel mutations associated with resistance. Genes containing mutations encode DNA gyrase subunit A, a hypothetical protein, an asparagine synthase, a sugar transamine/perosamine synthetase and others. Structural modeling performed on these proteins provides insights into the potential function of these proteins and how they might contribute to Cipro resistance mechanisms.

## Introduction

Ciprofloxacin (Cipro) is a broad-spectrum bactericidal fluoroquinolone antibiotic effective against many Gram-positive and Gram-negative bacteria. Its known mode of action is to bind to DNA topoisomerases involved in bacterial DNA replication, resulting in multiple double-stranded breaks in the bacterial chromosome. Studies of naturally-occurring mutations in several Gram-positive and Gram-negative pathogens that result in Ciprofloxacin resistance show that amino acid substitutions within the quinolone resistance-determining regions (QRDRs) of the *gyrA* and *parC* (and, in some cases, *gyrB* and *parE*) genes play crucial roles in resistance to this and other quinolone compounds. Cipro resistance in *B*. *anthracis* is associated with single nucleotide polymorphisms (SNPs) in *gyrA* and *parC* ([1] and our own unpublished results) but may also result from changes in either the structure or expression of multi-drug efflux pumps that actively remove antibiotics from microbial cells [2]. A single mutation within either a topoisomerase or an efflux pump gene or its regulatory region may be sufficient to make *B*. *anthracis* resistant to low Cipro concentrations. However, a combination of mutations is apparently required to confer resistance to higher antibiotic concentrations (19). Recent studies of *B*. *anthracis* ([3] and our own unpublished results) also identified mutations associated with Cipro resistance in TetR-type transcriptional regulator genes. Point mutations in *gyr*A and *mar*A associated with multi-drug and Cipro resistance have been observed in *Yersinia pestis* [4, 5] though they likely represent a minor fraction of the mutations that confer antibiotic resistance in this species.

While unintended selection of naturally occurring antibiotic resistant mutants through antibiotic overuse is a long-standing public health issue, a more recent concern is the possibility that hostile individuals or organizations could engineer resistant strains deliberately. These strains could be created either by targeted introduction of resistance elements or by selection of spontaneous mutants. Methods of inserting genetic material have been developed for a number of microbes including *B*. *anthracis*, *Y*. *pestis*, *F*. *tularensis* and *B*. *pseudomallei* [6–8]. It is therefore feasible that, by targeting genes that function in microbes closely related to specific threat agents, threat agent isolates that are resistant to therapeutically important antibiotic concentrations can be developed. Knowing which genes or combinations of genes are modified in Cipro resistant isolates, rapid assays can be developed that would detect these changes very quickly. These assays can be used to analyze the antibiotic resistance profiles in order to properly treat the exposed individuals as rapidly as possible. Moreover, such information would be valuable for forensic analysis to determine possible association with a suspected biowarfare or bioterrorism activity.

Unintended selection has resulted in a wide range of antibiotic resistant, clinically important pathogens. Most antibiotic resistance mechanisms fall into one of three classes: (1) Resistance based on changes in the structure of proteins targeted by the antibiotics, such as when changes in genes encoding components of topoisomerases change the shape of the sites where Cipro ordinarily binds to them; (2) resistance based on acquisition or increased expression of proteins that directly act on the antibiotic molecule, e.g. of β-lactamase enzymes that break down penicillins; and (3) resistance based on acquisition or upregulation of energy-dependent efflux pumps that actively remove antibiotics from the bacterial cells, such as the Bmr and Blt multi-drug transporter proteins of *Bacillus subtilis* (1). Efflux pumps are very common in Gram-negative bacteria, are often poorly characterized, and can result in co-resistance to several antibiotics. All three types of resistance mechanisms may be chromosomally encoded or may be acquired on extra-chromosomal elements.

Studies have shown that naturally occurring *F*. *tularensis* strains are susceptible to streptomycin, gentamicin, doxycycline, chloramphenicol and quinolones, and have heterogeneous susceptibility to erythromycin [9–11]. While *F*. *tularensis* can acquire Cipro resistance under selective pressure, the mechanisms of Cipro resistance in *F*. *tularensis* are not well understood. We selected for survival of *F*. *tularensis* LVS isolates in the presence of increasing Cipro concentrations, then compared whole genome sequences of resistant and related sensitive isolates to identify mutations likely to be found in *F*. *tularensis* subjected to Ciprofloxacin selective pressure. We performed whole genome analysis using a combination of two methods to assess the relative strengths of each platform for mutation detection. First, we developed a comparative genome hybridization (CGH) tiling microarray for *F*. *tularensis* LVS, with successive probes overlapping by at least 85% of their length, and performed two-color hybridizations of each resistant isolate together with the parent LVS strain. We then used Illumina next generation shotgun sequencing to generate large numbers of short sequence reads for each isolate, with more than 200X coverage over the entire genome. Here we describe the mutations found by these experiments and report the results of protein structure analyses to elucidate the underlying resistance mechanisms.

## Methods

### Selection of Cipro resistant mutants

A parental avirulent *F*. *tularensis* subsp. *holartica* LVS strain was provided by the CDC. *F*. *tularensis* LVS culture was streaked onto a Mueller Hinton broth (MHB) agar plate (enriched with Proteose Peptone, NaCl_2_, Bovine serum, D-(+) Glucose, Ferric Pyrophosphate, and Iso-Vitalex). The wild-type Ciprofloxacin minimum inhibitory concentration (MIC) value was determined for *F*. *tularensis* LVS by picking a single colony to inoculate 2 mL enriched MHB and incubating overnight at 37°C, 180 rpm. A subculture containing 2 mL enriched MHB was inoculated with 200 μL of the overnight culture and incubated at 37°C, 180 rpm to an optical density at 600 nm of 0.8. A Cipro E-test (BioMerieux) was applied to an enriched MHB agar plate swabbed for full coverage with the *F*. *tularensis* LVS subculture, and the E-test plate was incubated overnight at 37°C in an atmosphere containing 5% CO_2_. An approximate Cipro MIC was determined to be 0.023 μg/mL for the wild-type *F*. *tularensis* LVS.

Cultures were prepared for first-round selections by inoculating each well of a 24 well bioblock containing 2 mL of enriched MHB with the same single *F*. *tularensis* LVS colony. The bioblock was covered with an airpore tape seal and incubated at 37°C, 180 rpm overnight. Fresh subcultures were prepared by adding 20 μL of each overnight culture to 2 mL enriched MHB. The subcultures were incubated at 37°C, 180 rpm for approximately 4–6 hours to an optical density at 600 nm of 0.8. Cell suspensions were concentrated by centrifugation at 4,000 *g* for 2 min. The supernatant was discarded and each cell pellet was suspended in the remaining 200 μL of enriched MHB. Each of the 24 suspensions was plated on enriched MHB agar plates containing 0.075 μg/mL Cipro (approximately three times the wild-type MIC value). These 24 first-round selection plates were incubated at 37°C, up to 72 hours in a CO_2_ enriched atmosphere. One Cipro resistant colony was picked from each plate into 2 mL enriched MHB containing 0.05 μg/mL Cipro (75% of the resistant concentrations) and incubated at 37°C, 180 rpm overnight. Subcultures were prepared by adding 20 μL of the passage culture that grew in the presence of Cipro to 2 mL enriched MHB without Cipro and incubating at 37°C, 180 rpm to an optical density at 600 nm of 0.8. These subcultures were used for MIC value determinations (as indicated above) and to prepare frozen stocks by adding 700 μL of the subculture to 300 μL sterile 80% glycerol followed by storage at -80°C. Second- and third-round selections using first-round resistant isolates were carried out by increasing Cipro concentrations to approximately three-fold the parent generation MIC values at each step. Approximately 10 second-round resistant isolates were collected following selection for resistance to a higher Cipro concentration for each of 24 first-round mutants (approximately 240 total), and up to five third-round resistant isolates were collected following exposure of each second round isolate to even higher Cipro concentrations producing approximately 1,000 Cipro resistant *F*. *tularensis* LVS isolates.

Resistant colonies were verified to be *F*. *tularensis* LVS by colony morphology and *F*. *tularensis*-specific PCR with a forward primer of: GGCTATATGATGGCATTTTTATTAG; and a reverse primer of: GATATATACCCATTATCGAACCATCC. Glycerol stock dilutions were used directly as templates for the PCR analyses.

### Whole genome tiling array design for *F*. *tularensis* LVS

Tiling arrays were designed for *F*. *tularensis* LVS using the NimbleGen 388K array platform, which supports probes of multiple lengths on the same array. We developed computational tools to design probes that tile across entire bacterial genomes while satisfying length, overlap and melting temperature (T_m_) constraints. By designing and hybridizing *F*. *tularensis* DNA to several test arrays, we determined that a length range of 32–40 nucleotides (nt) provided optimal sensitivity and specificity; reference genomic DNA did not consistently bind to probes shorter than 32 nt, while probes longer than 40 nt did not discriminate well between perfect match targets and targets containing SNPs (data not shown). Individual probe lengths were selected to minimize the overall variation of melting temperatures, given the allowed length range of 32–40 nt. A T_m_ range of 74±3°C was selected, based on GC content of the *F*. *tularensis* LVS genome and a median probe length of 36 nt. Melting temperatures were calculated using Unafold [12] which employs accurate nearest neighbor thermodynamic predictions.

Probes were tiled with an overlap of 85% (every 5–6 nt) across the sequences of the *Francisella tularensis* subsp. holarctica LVS chromosome (GenBank gi number 89143280) and plasmids pOM1 from *F*. *tularensis* LVS (gi number 10954617), pFPHI01 from *F*. *philomiragia* subsp. philomiragia strain ATCC 25017 (gi number 167626220), and pFNL10 from *F*. *tularensis* subsp. novicida strain F6168 (gi number 32455353). There were a total of 363,359 unique tiled probe sequences on the array. Every seventeenth probe was replicated on the array. We included 3,494 probes containing randomly generated sequences, matching the length and GC% distributions of the tiled probes as negative controls for assessing the distribution of background signals.

### Microarray hybridization of mutant and reference DNAs

Genomic DNAs from wild type and Cipro resistant isolates were isolated using a Promega Wizard™ genomic DNA purification kit. DNA labeling and hybridization were performed as described in [13] with the following modifications. The reference LVS DNA was labeled with Cy3-labeled random 9-mers and the DNA from the Cipro resistant isolates was labeled with Cy5-labeled random 9-mers. Two μg of the Cy3 labeled reference DNA and Cy5-labeled DNA from a Cipro resistant isolate were hybridized to the same array. Hybridization was for 17 hours at 42°C temperature. Following hybridization, arrays were washed, then scanned using an Axon 4000B scanner (Molecular Devices, Sunnyvale, CA) at 5 μm resolution. Excitation wavelengths of 532 nm and 635 nm were used to detect Cy3 and Cy5 hybridization, respectively. Array images were saved as TIFF files. NimbleScan software 2.4 (Roche Diagnostics) was used to compute the probe fluorescent intensities from TIFF images and overlay them to pair file reports (text files with the signal intensities from the array). The pair reports were used for statistical analysis of microarray data.

### Statistical analysis of sequence changes from tiling microarrays

An algorithm called TAPS (Tiling Array Polymorphism Sensor) was developed to analyze data from the two-color hybridizations. The TAPS algorithm is based on a thermodynamic model that predicts the effect of mutations on probe-target hybridization affinities, and estimates the likelihood of a mutation at every reference genome position, given the intensities of all probes overlapping the position, The algorithm superficially resembles the “SNPscanner” algorithm of Gresham et al [14], but requires fewer training parameters (70 vs. 4608), and is less susceptible to over-fitting. It can also analyze two-color data sets, and is not restricted to Affymetrix array designs.

The TAPS algorithm models the effect of a SNP on the intensity of an overlapping probe as a function of several variables: the reference channel probe intensity, the position of the SNP in the probe sequence, the base substitution relative to the reference genome, and the two perfect-match bases on either side of the SNP locus. We assume that probe intensity decreases as the free energy of hybridization increases (becomes less negative), and that the free energy ΔG is a sum of contributions from aligned pairs of nearest-neighbor (NN) nucleotides. A SNP in the target sequence increases the free energy by replacing two perfect-match NN pairs with pairs having a single mismatch. For example, a mutation that changes the sequence AGC to ATC replaces the perfect match pairs AG/TC and GC/CG with the mismatch pairs AT/TC and TC/CG. Since our tiling array only has probes for the reference genome sequence, it does not provide information about the specific base substitution in the target genome. However, we can predict the *average* effect of the three possible substitutions at the central base of a particular base triplet. To estimate these average mutation effects for the different base triplets, we performed experiments in which labeled DNA from the reference LVS strain was hybridized to an array, together with a differentially labeled DNA from a different *F*. *tularensis* strain of known sequence (subspecies tularensis strain Schu S4 or subspecies novicida strain U112), and thus, with known sequence variations relative to the LVS strain. S1 Fig shows the distributions of log intensity ratios for probes overlapping known sequence variations between the LVS and Schu S4 strains, for an array hybridized to these two strains. The distributions are shown as a box plot, with probes grouped by the reference triplet centered at the SNP locus. As expected, SNPs affecting a triplet with a central G or C base have a stronger effect on average than those replacing an A or a T.

The TAPS model also includes a multiplicative position effect, in which SNPs aligning near the middle of a probe cause larger intensity drops than SNPs aligned near the ends, especially the 3’ region closest to the array surface. We expected to see this positional effect based on our earlier work with virulence gene arrays [13]. S2 Fig shows a typical profile of intensity change vs. SNP position, for the same Schu S4 vs. LVS array used in S1 Fig. Each column in this plot represents the distribution of log intensity ratios between the Cy3 (LVS) and Cy5 (Schu S4) channels, for probes overlapping a Schu S4 variation at a given position in the probe; the central bar represents the range from the 25^th^ to the 75^th^ percentiles. We see that, on average, the intensity drop is almost two-fold when a SNP affects the nucleotides binding near the middle of the probe, but is reduced to zero at either end.

Even in the absence of SNP effects, probe intensities will differ between the two channels due to dye effects, scanner bias and noise. To correct for these effects, each pair of intensities (*y*_*ref*_, *y*_*mut*_) was transformed into the log ratio (M) and log geometric mean (A):

$$M=\text{log}\frac{y_{mut}}{y_{ref}}$$

$$A=\frac{1}{2}(\text{log}\;y_{ref}+\text{log}\;y_{mut})$$

A semi-parametric regression model was fitted using the *M* vs. *A* data for all probes:

M=μ(A)+ε(A)

in which the error term *(A)* has mean 0 and variance ^*2*^*(A)*, and *μ(A)* and *σ*^*2*^*(A)* are smooth mean and variance functions. The functions *μ(A)* and *σ*^*2*^*(A)* were fit to the *M* and *A* values for all probes on each array, using regression on cubic splines to fit *μ(A)*, and a smoothing spline on binned squared residuals to fit *σ*^*2*^*(A)*. Since SNPs only affect a small fraction of probes on the array, the fitted *μ(A)* closely approximates the mean function for perfect match probes (those not overlapping variations between the reference and target strains).

To model the effect of a free energy change *ΔΔG* = *ΔG*_*mut*_*− ΔG*_*ref*_ on the log intensity ratio, we assume that the probe DNA oligomers within an array feature can be in one of three states: unbound, bound to target DNA from the mutant strain, or bound to target DNA from the reference. At thermodynamic equilibrium at temperature *T*, the fraction of oligomers bound to mutant DNA is given by the Boltzmann equation:

$$\theta_{mut}=\frac{e^{-∆G_{mut}/RT}}{1+e^{-∆G_{mut}/RT}+e^{-∆G_{ref}/RT}}$$

where *R* is the gas constant; a similar equation holds for the fraction of oligomers bound to reference DNA, *θ*_*ref*_. It follows that

$$\text{log}\frac{\theta_{mut}}{\theta_{ref}}=-\frac{∆∆G}{RT}$$

Since the probe intensity for each dye at concentrations well above background and below saturation scales with the fraction of oligomers bound to target labeled with that dye, we expect the SNP effect on the log intensity ratio to be proportional to *ΔΔG*. Therefore, for probes overlapping SNPs, our semi-parametric regression model is modified to include a term for the SNP effect:

$$M_{obs}=\mu (A_{obs})+w\;∆∆G+\varepsilon (A_{obs})$$

where *w* is a proportionality constant (typically < 0) and the noise term (*A*) is assumed to be Gaussian with mean 0 and the same variance ^*2*^*(A)* as was estimated for perfect match probes. The free energy effect *w ΔΔG* is modeled as a product of triplet and position effects:

$$w\;∆∆G=\beta_{\tau }h(x)$$

where *τ* indexes the triplet and *x* is the position of the SNP within the probe, as a fraction of the probe length. The position effect *h*(*x*) is approximated by a polynomial function of degree 5:

$$h(x)=\sum_{j=0}^{5}a_{j}x^{j}$$

The triplet effects are assumed to be equivalent for reverse complements, so there are 32 *β*_*τ*_ parameters and six *α*_*j*_ parameters to be fit. Note that the proportionality constant *w* has been absorbed into the triplet effects. The model parameters were fit to data from the experiments described above, in which arrays were hybridized to DNA from *F*. *tularensis* strains of known genome sequence, and thus with SNPs at known positions relative to the reference LVS genome. To make the parameters identifiable, we scaled the coefficients *α*_*j*_ so that *h*(0.5) = 1.

To apply the model to data from target strains of unknown sequence, we computed a log likelihood ratio test statistic for every position *z* in the reference genome. Let *P*(*z*) be the set of probes overlapping position *z*, and let *M*_*i*_ and *A*_*i*_ be the log intensity ratio and average for probe *i*. The semi-parametric regression model given above leads to the following expression for the log likelihood:

$$\text{log}\;L(z)=-\sum_{i\in P(z)}\frac{{\left(M_{i}-\mu (A_{i})-w\;∆∆G_{i}\right)}^{2}}{2\sigma^{2}(A_{i})}$$

Under the null hypothesis that there is no SNP at position *z*, then *ΔΔG*_*i*_ *=* 0 for all probes in *P*(*z*), and the log likelihood is given by:

$$\text{log}\;L_{0}(z)=-\sum_{i\in P(z)}\frac{{\left(M_{i}-\mu (A_{i})\right)}^{2}}{2\sigma^{2}(A_{i})}$$

Under the alternative hypothesis that there is a SNP at position z, *ΔΔG*_*i*_ was computed for each probe using the fitted model parameters, leading to a different log likelihood value log *L*_*alt*_(z). The log likelihood ratio test statistic is simply:

$$\text{log}\;LR(z)=\text{log}\;\frac{L_{alt}(z)}{L_{0}(z)}=\text{log}\;L_{alt}(z)-\text{log}\;L_{0}(z)$$

**Fig 1:**
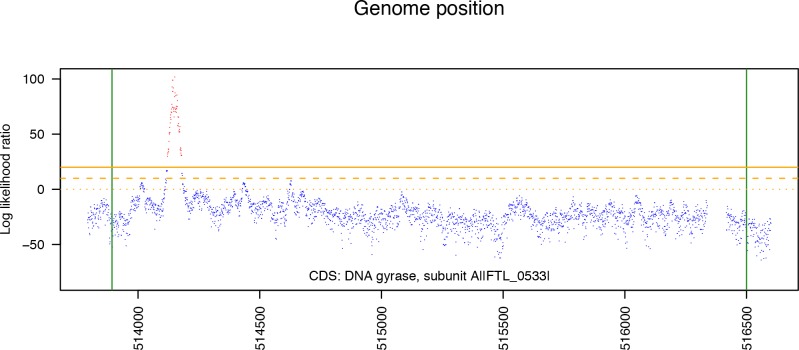
The test statistic values for a short region of the DNA gyrase A gene in one of the Cipro resistant *F*. *tularensis* isolates. The log likelihood ratio has a clear peak in this region. Candidate SNP positions were identified by looking for regions of the genome where the log likelihood ratio exceeds a fixed threshold.

To identify candidate SNP loci, we computed log *LR(z)* for every position *z* in the reference genome and compared it to a threshold value, which we selected by analyzing data from the test arrays hybridized to DNA from *F*. *tularensis* strains with SNPs at known positions relative to the LVS strain, and choosing the threshold that gave the best tradeoff between false positive and false negative error rates. This threshold was 20 for the *F*. *tularensis* arrays. Typically SNPs were characterized by a contiguous series of position values with log *LR* scores above the threshold. The most likely SNP location within the series was identified by the position with the maximum score. As an example, Fig 1 plots the test statistic values for a short region of the DNA gyrase A gene in one of the Cipro resistant *F*. *tularensis* LVS isolates. The log likelihood ratio has an obvious peak in this region.

### Illumina sequence data generation and quality control

Illumina paired end libraries were prepared from 1 μg of genomic DNA from each of eleven third round Cipro resistant isolates, for the purpose of single-end sequencing on the Genome Analyzer IIx. Briefly, the gDNA was fragmented, ends repaired, A’ tagged, ligated to adaptors, size-selected and enriched with 13 cycles of PCR. Each library was assigned one lane of a flow cell to undergo cluster amplification and sequencing on the Genome Analyzer IIx, and 36 cycles of single-end sequence data were generated. One lane of paired end 51 cycle sequence data were generated for *F*. *tularensis* LVS Cipro resistant isolate 1:1:5. The resulting sequencing reads were filtered using the default parameters of the Illumina QC pipeline (Bustard + Gerald).

As an additional quality control step, all reads were analyzed using the PIQA pipeline [15]. This pipeline examines genomic reads produced by Illumina machines and provides tile-by-tile and cycle-by-cycle graphical representations of cluster density, quality scores, and nucleotide frequencies. This method allows easy identification of defective tiles, mistakes in sample/library preparations and abnormalities in the frequencies of appearance of sequenced genomic reads. All reads were determined to be of sufficient quality to proceed with subsequent analysis. The amount of sequence data generated for each sample is indicated in Table 1.

**Table 1:** Illumina sequence data summary.

Isolate ID	Total sequenced amount (Mb)	Read size	Total # of reads	# of unique reads	% unique reads	Median coverage^a^
*F*. *tularensis* LVS 1:1:5	1,486	51	29,128,996	7,957,310	27.32%	711 x
*F*. *tularensis* LVS 5:8:3	730	36	20,269,065	4,626,044	22.82%	326 x
*F*. *tularensis* LVS 8:7:2	718	36	19,944,491	4,863,025	24.38%	317 x
*F*. *tularensis* LVS 11:4:3	748	36	20,777,320	4,365,901	21.01%	332 x
*F*. *tularensis* LVS 12:3:4	677	36	18,813,642	4,580,189	24.35%	266 x
*F*. *tularensis* LVS 14:6:5	690	36	19,161,169	4,259,430	22.23%	274 x
*F*. *tularensis* LVS 15:6:5	580	36	16,120,185	4,297,079	26.66%	275 x
*F*. *tularensis* LVS 16:10:2	607	36	16,847,563	4,004,713	23.77%	302 x
*F*. *tularensis* LVS 18:5:2	511	36	14,199,956	3,526,148	24.83%	211 x
*F*. *tularensis* LVS 21:8:2	607	36	16,852,775	3,707,584	22.00%	284 x
*F*. *tularensis* LVS 23:2:4	573	36	15,904,934	3,626,329	22.80%	259 x
*F*. *tularensis* LVS WT	590	36	16,400,385	3,764,767	22.96%	282 x

^a^Median coverage of reference genome (*F*. *tularensis* NC_007880) by reads mapped with up to 1 mismatch (insertion/deletion/substitution).

### Mapping and identifying candidate mutations

The sequence reads from each of the samples were mapped with up to 1 mismatch to the reference *F*. *tularensis* LVS genome (RefSeq accession NC_007880). To avoid uncertainty associated with identifying mutations in repeatable parts of the reference genome, for each position in the reference sequence a *uniqueness score* based on the subsequences covering this nucleotide was determined. Specifically, the copy number of each subsequence of size 36 (the length of reads used in sequencing) present in the reference genome was first calculated; the *uniqueness score* of each position in the reference genome was then defined as the total number of subsequences (factoring in the copy number) which covered this position. For example, in this metric, the score of 36 will appear only if each subsequence covering a given nucleotide is unique in the reference; higher scores indicate that one or more subsequences are present in the reference in several copies. 94.11% (1,784,242 bases) of the *F*. *tularensis* LVS (NC_007880) reference genome has a uniqueness score of 36. Mutations in these positions can be detected without the ambiguity caused by the presence of repeatable regions.

A given position is predicted to contain a mutation if: (1) the number of reads confirming the mutation on each strand exceeds the *minimum count threshold–*ensuring that only positions that achieve the minimum required coverage are considered, and (2) the proportion of reads confirming a mutation out of all the reads covering a given position exceeds a *ratio threshold–*ensuring that only mutations that have the minimum required support are identified. As a compromise between mutation detection sensitivity and false discovery rate, the *minimum count threshold* was set at 10% of the median of the nucleotide-by-nucleotide coverage for each sample, and the *ratio threshold* was set at 30% of the total coverage on a per-nucleotide basis. In the present analysis, mutations confirmed on both strands (if the number of reads supporting the mutation exceeds the *minimum count threshold* on each of the strands separately) are distinguished from mutations for which such a condition was met on only one strand. In the case of insertions, the mapping process results in the association of both perfect matches (PM) and insertions to the same location on the reference genome. Thus different *ratio threshold* criteria are used to detect different types of mutations at a given genome position. The criterion for detecting a substitution of base *B* for the reference base is:

$$\frac{SubB^{+}+SubB^{-}}{{PM}^{+}+{PM}^{-}+{Del}^{+}+{Del}^{-}+{SubACTG}^{+}+{SubACTG}^{-}}\geq ratio\;threshold$$

The criterion for detecting a deletion is:

$$\frac{{Del}^{+}+{Del}^{-}}{{PM}^{+}+{PM}^{-}+{Del}^{+}+{Del}^{-}+{SubACTG}^{+}+{SubACTG}^{-}}\geq ratio\;threshold$$

The criterion for detecting an insertion of base *B* on the plus strand is:

$$\frac{{InsB}^{+}+{InsB}^{-}}{{Del}^{+}+{Del}^{-}+{SubACTG}^{+}+{SubACTG}^{-}+{InsACTG}^{+}+{InsACTG}^{-}}\geq ratio\;threshold$$

In the numerators of the above formulas, *SubB*^*+/-*^, *Del*^*+/-*^, and *InsB*^*+/-*^ stand for the numbers of reads confirming a substitution, deletion, or insertion, respectively, mapping to the genome strand indicated by the superscript. For substitutions and insertions, *SubB*^*-*^ and *InsB*^*-*^ indicate the numbers of reads mapped to the minus strand in which the base complementary to *B* is substituted or inserted. In the denominators, the variables *PM*, *SubACTG*, and *InsACTG* respectively indicate the numbers of reads confirming a perfect match (PM), a substitution of any base, or an insertion of any base, at the genome position of interest.

While paired end data was generated, the reads were decoupled and a single-end read assembly (using in-house algorithms) was performed on each of the sequence data sets. These contigs are shorter in length than contigs obtained with paired end data, but in general have fewer errors. Each mutation identified in each sample was confirmed to be present on the contigs assembled for that sample. Mutations (including insertions, deletions, and substitutions) that pass both thresholds and appear on both strands are less likely to be sequencing read generation or mapping artifacts. Mutations that only appear on one strand and cannot be verified on the opposite strand (something that is not common, given sufficient coverage), such as insertions, other than ‘G’ after ‘G’, ‘C’ after ‘C’, ‘A’ after ‘A’, and ‘T’ after ‘T’ are likely artifacts of sequencing/mapping (false positives) or positions in the genome that did not have sufficient coverage to be verified on both strands.

### PCR and Sanger sequencing confirmation of Cipro-resistant mutants

To confirm mutations identified by tiling microarray and Illumina sequencing, PCR oligonucleotide primers were designed using Primer3™ [16] to amplify *F*. *tularensis* LVS genome-specific sequences surrounding the locus where the mutations were identified. In addition to round 3 Cipro-resistant isolates, PCR and sequencing reactions were also performed on round 1 and 2 isolates to identify the selection step in which each mutation occurred. PCR was performed using Promega PCR reagents. Sanger sequencing was performed using ABI3730 DNA analyzers at the DOE Joint Genome Institute in Walnut Creek, CA or at Elim Biopharmaceuticals, Inc (Hayward, CA).

### Analysis of the impact of the mutations on protein structure and function

The automated homology modeling system AS2TS [17] was used with other computational tools (http://proteinmodel.org) to construct and analyze structural models for all *F*. *tularensis* LVS proteins (listed in Table 2 and S4 Table). Created structural models were analyzed to assess the possibility of conformational changes implied by the observed mutations, and to estimate the level of possible sequence variability in identified structurally conserved regions. Structure alignments were calculated using the program LGA (Local Global Alignment) [18] and evaluation of detected structural similarities between LVS proteins and related structures from Protein Data Bank (PDB) was performed by StralSV sequence/structure variability evaluation system [19]. StralSV identifies all structurally similar protein structure fragments in the PDB for any given structural motif, evaluates calculated structure-based alignments between the query motif and the fragments, and quantifies observed sequence variability at each residue position. The output from the system enables rapid identification of invariant residues (often those essential to protein function) and unusual variants, and predictions about natural or engineered mutations that are not yet observed in current sequence databases. Results from the StralSV analysis allowed us to characterize observed mutation points by assigning their location on the protein (e.g. buried, exposed, within an active site), and to identify other proteins (sometimes from more distant organisms) in which a similar structural motif with a given substitution was observed and characterized.

**Table 2:** Genes in Cipro resistant *F*. *tularensis* LVS isolates containing mutations identified both by Illumina sequencing and SNP microarray. The reference genome *F*. *tularensis* NC_007880 was used to determine the reference genome position. The lists of identified amino acid diversities at the given mutation points observed in the corresponding positions in homologous proteins are provided in the “Amino acid change” column.

Gene annotation	Mutation and codon context	Amino acid change (AA diversity in homologous proteins in order from most to least frequent)	Cipro resistant isolates containing this mutation	Reference Genome Position
Hypothetical membrane protein [FTL_0073] complement(70687..71667)	625C->A GCTTCATGG	E-209->stop (E,F)	8:7:2	71,043
TPR (tetratricopeptide repeat) domain protein [FTL_0204] region(203990..204988)	344G->T GAGCGAGCT	R-115->L (R,Q,K,M,E,A,I,L)	11:4:2	204,333
Hypothetical protein [FTL_0439] region(406452..408107)	16A->T ATTTAAAAA	K-6->stop (K,R,L,S)	15:6:5	406,467
Hypothetical protein [FTL_0439] region(406452..408107)	588C->A CGTACTCTG	Y-196->stop (Y,F,S,D,K,A)	21:8:2	407,039
Hypothetical protein [FTL_0439] region(406452..408107)	1249G->T TATCGATAC	D-417->Y (D,N,G,S,Q,E,R,A,V,Y)	1:1:5 11:4:3 12:3:4 18:5:2	407,700
Hypothetical protein [FTL_0439] region(406452..408107)	1250A->G ATCGATACC	D-417->G (D,N,G,S,Q,E,R,A,V,Y)	23:2:4	407,701
Glucosamine—fructose-6-phosphate aminotransferase [FTL_0454] region(429717..431555)	1526C->A GGTCCTCTA	P-509->H (P,T,S,A,I,V,F,N,G,D,H)	21:8:2	431,242
DNA gyrase, subunit A [FTL_0533] region(513894..516500)	248C->A GATACAGCT	T-83->K (S,T,A,Q,L,I,N,F,V,M,G,Y,R,K)	12:3:4 14:6:5 21:8:2	514,141
DNA gyrase, subunit A [FTL_0533] region(513894..516500)	248C->T GATACAGCT	T-83->I (S,T,A,Q,L,I,N,F,V,M,G,Y,R,K)	1:1:5 5:8:3 8:7:2 11:4:3 16:10:2	514,141
DNA gyrase, subunit A [FTL_0533] region(513894..516500)	259G->A TTACGATAC	D-87->N (D,E,G,F,M,N,L,Q,Y)	5:8:3	514,152
DNA gyrase, subunit A [FTL_0533] region(513894..516500)	259G->T TTACGATAC	D-87->Y (D,E,G,F,M,N,L,Q,Y)	15:6:5 16:10:2 11:4:3 18:5:2 23:2:4	514,152
Conserved hypothetical protein, pseudogene [FTL_0551] region(533031..533885)	809G->T TTCAGGACA	R-270->M (R)	5:8:3	533,839
UDP-glucose/GDP-mannose dehydrogenase [FTL_0596] region(582479..583789)	274G->T ACCAGTTAA	V-92->F (I,V,L,T,A,P,F)	16:10:2	582,752
asparagine synthase [FTL_0600] region(587103..588989)	1194G->T CTAAGTTAC	K-398->N (R,K,A,P,S,Q,G,V,N)	15:6:5	588,296
asparagine synthase [FTL_0600] region(587103..588989)	1807G->A GCAAGAGCA	E-603->K (E,Q,D,A,N,R,K)	11:4:2	588,909
sugar transamine/perosamine synthetase [FTL_0601] region(588982..590064)	206C->A AGAGCGTTA	A-69-> (A,S,T,V,L,G,I,N,C,D,M,F,K,Q,E)	8:7:2	589,187
sugar transamine/perosamine synthetase [FTL_0601] region(588982..590064)	330C->T TAGACGTCT	D-110->D (D,N,S)	15:6:5	589,311
Glycosyltransferase [FTL_0604] region(592271..593131)	317C->A CAGGCTGAT	A-106->D (V,A,I,L,Y,S,M,F,N,K,H,T,C,D)	12:3:4	592,587
Conserved hypothetical protein [FTL_1107] region(1052313..1053695)	1002G->T CTCAGTTGA	Q-334->H (Q,A,L,E,S,K,W,N,Y,D,V,I,P,M)	16:10:2	1,053,314
Intergenic between [FTL_1327] type II-B CRISPR associated Cas9/Csx12 and [FTL_1328] outer membrane associated proteins region(1263690..1264351)	473T->A ACATTCTCT	Not applicable	15:6:5	1,264,162
DNA gyrase subunit B [FTL_1547] complement(1476400..1478811)	1394G->T TTGAGAACC	S-464->Y (S,N,A,Y)	18:5:2	1,477,418
Efflux protein, RND family, MFP subunit [FTL_1671] region(1604054..1605427)	435C->A TTAGCGAAA	S-145->R (D,S,T,K,I,V,L,R)	15:6:5	1,604,488
Hypothetical protein [FTL_1945] complement(1874376..1875031)	504A->T GTACAAAAA	C-169->S (F,V,L,C,R,Y,W,T,I,E,A,S)	15:6:5	1,874,528

### Testing multi-drug resistance properties of the *F*. *tularensis* LVS Cipro resistant mutants

A total of 148 third round *F*. *tularensis* LVS Cipro-resistant isolates were screened for resistance to other antibiotic drugs including amoxicillin w/clavulanic acid, ampicillin, carbenicillin, doxycycline, erythromycin, gentamicin, nalidixic acid, rifampin and streptomycin. Cells were plated onto enriched Mueller Hinton broth agar plates, and Sensi-discs (BD, Franklin Lakes, NJ) impregnated with the aforementioned antibiotics were applied to the surface. Plates were incubated at 37°C with 5% CO_2_ for two days. Isolates were determined to be resistant if the zone of inhibition was at least 1 mm less than the zone of inhibition of the wild type. MIC values for amoxicillin with clavulanic acid and ampicillin were determined with an amoxicillin with clavulanic acid (XL) E-test strip or ampicillin (AM) E-test strip (BioMerieux).

## Results

### Minimum inhibitory Cipro concentrations for *F*. *tularensis* LVS Cipro resistant isolates

Following three rounds of selection by exposure to increasing Cipro concentrations, 289 Cipro resistant *F*. *tularensis* LVS isolates were collected and characterized. This included 28 first round isolates, 94 second round isolates and 148 third round isolates. The MIC value for the original Cipro-sensitive LVS strain was 0.023μg/mL. MIC values ranged from 0.25 to 1 μg/mL for first round resistant isolates, 0.5 to 16 μg/mL for second round isolates, and from 6 to greater than 32 μg/mL (the limit of the Ciprofloxacin E-test) for isolates collected following the third round of selection. The Cipro resistant isolates grew much more slowly than the Cipro sensitive isolates. The full set of MIC measurements is given in S1 Table. Each isolate is identified with a one, two or three-digit code indicating its lineage; for example, round 3 isolate 15:6:5 was derived from round 2 isolate 15:6, which was derived from round 1 isolate 15.

### Analysis of microarray data for *F*. *tularensis* LVS Cipro resistant isolates

We tested the wild type LVS strain and 11 third round Cipro resistant *F*. *tularensis* LVS isolates using the *F*. *tularensis* LVS tiling array. Forty-eight distinct mutations from a total of 15 gene regions were identified in two or more of the 11 Cipro resistant isolates. The list of mutations identified by the microarray from two or more clones is shown in S2 Table.

The isolate with the largest number of mutations identified by the microarray was isolate 15:6:5, with 21 candidate mutations. Mutations at six identical positions were each found in two or more of the 11 Cipro resistant isolates tested. There is one mutation at position 514,141 in the *F*. *tularensis* LVS genome in *gyrA*, occurring in four isolates, plus five other mutations, each occurring in two isolates. Array probes are tiled every 5 to 6 bases along the LVS genome. Therefore, the actual position of each mutation could be anywhere within 5 bases of the location having the peak log likelihood ratio. Therefore, identified mutations in different isolates whose estimated positions differ by 10 or fewer bases may potentially have resulted from SNPs at the same position.

A six base deletion in clone 18:5:2, in gene FTL 0598 (Membrane protein/O-antigen protein) was identified by microarray, but missed by Illumina sequencing. The mutation analysis used in sequencing analysis was targeting single mutations, not multiple deletions, which could result in mis-detection by sequencing. Mutations identified by the microarray that were also found by Illumina sequencing are listed in Table 2.

### Analysis of Illumina sequencing data for *F*. *tularensis* LVS Cipro resistant isolates

We analyzed the DNA from the wild type and 11 third round Cipro resistant *F*. *tularensis* LVS isolates using Illumina sequencing. Mutations were identified by comparison to the published reference LVS sequence. Three mutations at positions 152,924 (C->A), 717,695 (T->G) and 1,713,576 (G->T) were identified in all Cipro resistant isolates and the parent wild-type isolate. These are most likely due either to errors in the published reference sequence, or to mutations that appeared during passaging of the original LVS culture prior to selection for Cipro resistance.

A total of 30 unique mutations from 20 genes, one 7 T insertion and one single T insertion were identified in the 11 third round Cipro resistant samples using Illumina sequencing. The location of the SNPs, the gene description, gene location, the number of reads and the percentage of reads containing the SNP are shown in S3 Table. The number of reads where the mutations were identified ranged from 80 reads in isolate 23:2:4 at position 851,357 to 842 reads at position 514,141 for isolate 1:1:5. Twenty-six SNPs were detected at >90% of all reads at that position. The 4 SNPs that were detected at <90% of reads at that position are 406,467 (isolate 15:6:5, 198 reads, 60.2% reads detected the SNP), 436,130 (isolate 5:8:3, 334 reads, 51.5% with SNP), 466,243 (isolate 14:6:5, 202 reads, 65.6% with SNP), 1,683,798 (isolate 1:1:5, 559 reads, 64.1% with SNP). There was an insertion of 7 Ts in isolate 23:2:4 at positions 578,478 to 578,484, all of which were detected at 100% of the reads. A single T insertion was identified at position 1,706,353 in all 11 isolates. Since the number of reads containing this insertion is low (from 6 to 40) and this insertion was found in all of the isolates tested, it is possible that this is a sequencing error. This insertion was not included in the final table of SNPs.

Two SNPs confirmed on one strand were identified at position 533,839 (G->T) in clone 5:8:3 and at position 850,695 (A->C) at clone 12:3:4 (not included in S3 Table). The SNP at position 533,839 is likely real since this mutation was also identified by microarray. This mutation is included in Table 2. The SNP at position 850,695 is likely an incorrect read because it is not confirmed in the contig and is found only in sequences at the edge of the reads containing the SNP. Cipro resistant sample 15:6:5 contained the highest number of SNPs with 11.

Mutations that were identified by Illumina sequencing and also confirmed by SNP microarray are shown in Table 2. There are a total of 23 mutations from 15 genes that are consistent between the two technologies. Mutations that were identified by Illumina and not confirmed by microarray are listed in S4 Table.

### Protein structural modeling to analyze the effects of the mutations

#### Mutations on FTL_0439, hypothetical protein

There are four SNPs identified in the genetic locus FTL_0439 in *F*. *tularensis* LVS isolates resistant to Cipro. Seven out of the 11 Cipro resistant isolates tested have at least one SNP in this gene, with the SNP at position 407,700 being the most common. The triplet that includes nucleotide 407,700 in the *F*. *tularensis* genome encodes Asp at amino acid position 417 in this hypothetic protein. Mutations found in Cipro resistant isolates would change this to Tyr. None of the first round resistant isolates has a mutation in this gene. Only one second round isolate has a mutation at position 407,700 suggesting that this mutation did not occur in the first round of selection and likely occurred in isolates prior to the third round of selection.

SNPs at two additional positions in FTL_0439 were identified. A mutation at position 406,467 would result in a change from a protein with a Lys at position 6 to a truncated protein as a result of a stop codon present at this position; The nucleotide normally present at position 407,039 encodes a Tyr at amino acid position 196 in this protein and is also changed to a stop codon when the observed mutation is present.

Results from the homology modeling suggest that FTL_0439 could be an outer membrane protein involved in ion transport. However, because the level of sequence identity between FTL_0439 and identified structural templates from PDB is very low, around 15%, a detailed structural analysis of the created models cannot be conducted with sufficient confidence.

#### Mutations in the DNA gyrase FTL_0533

Two SNPs in the *gyrA* gene (genome locations 514,141 and 514,152) were found in all third round Cipro resistant isolates subjected to microarray and Illumina analyses. Additional Sanger sequencing confirmed that all third round Cipro resistant isolates analyzed have one or both of these SNPs in *gyr*A. All first round isolates analyzed by Sanger sequencing contain a SNP at either 514,141 or 514,152, but none of the first round isolates has SNPs at both locations. As expected, all second round isolates tested also have a SNP at either position 514,141 or 514,152 and approximately one-third of the second round isolates tested contain SNPs at both locations. This suggests that one SNP occurs early in the selection process, and the second SNP arises during second or third round selection. Either mutation will result in a change of an amino acid in the DNA gyrase encoded by FTL_0533. There is normally a Thr encoded by the wild-type gene but detected SNPs at position 514,141 would change this to Lys or Ile. The DNA sequence that includes position 514,152 normally encodes an Asp at position 87 in the DNA gyrase gene. Detected SNPs caused a change at this location to Asn or Tyr.

**Fig 2:**
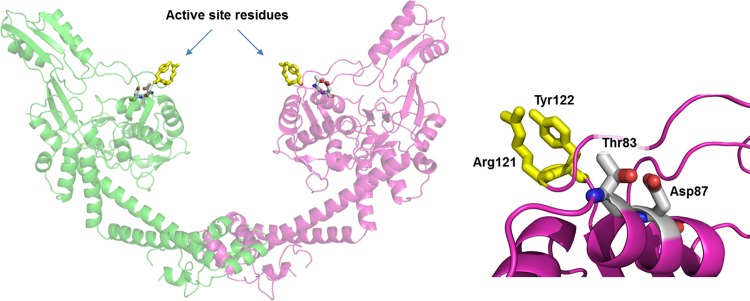
Structural model of the open dimeric conformation of DNA binding/cleavage domain of *Gyr*A from FTL_0533. Left plot: the active site residues essential for DNA cleavage Arg121 and Tyr122 are shown in yellow sticks. Right plot close-up of the region outlined by the rounded rectangle shows location of the mutated positions Thr83 and Asp87.

A protein structural model of FTL_0533 was constructed to identify the potential structural and functional effects of the two mutations at positions 83 and 87. The N-terminal domain of GyrA from *E*. *coli* (PDB chain: 2wl2_B) was used to construct the homology model. The percentage of amino acid sequence identity between FTL_0533 and 2wl2_B is 65%. Structural analysis of created models showed that the mutations at Thr83 and Asp87 are located on the fragment 83–90 which is a short helical region in close proximity to the active site residues Arg121 and Tyr122 (Fig 2). A recent study of the gyrA crystal structure from *E*. *coli* showed that the substitutions in the region 81 to 84 and at position 87 in *F*. *turlarensis* would likely impact drug binding [20].

#### Mutations in FTL_0600 encoding asparagine synthase

Two mutations were identified in FTL_0600 at nucleotide positions 588,296 and 588,909 from two of the 11 isolates, 15:6:5 and 11:4:2. The triplet that includes nucleotide 588,296 normally encodes Lys at amino acid position 398 in this protein. The mutation observed results in a change to Asn. The triplet that includes nucleotide 588,909 normally encodes a Glu at position 603 in the protein and the observed mutation results in a Lys at this position. A structural model of FTL_0600 was constructed based on predicted similarity to the crystal structure of Asparagine synthase B from *E*. *coli* (PDB chain: 1ct9_A). The level of sequence identity between the protein encoded by FTL_0600 and the closest structural templates from PDB is 22%, hence a detailed structural analysis of the created models cannot be conducted with sufficient confidence.

#### Mutations in FTL_0601 encoding sugar transamine/perosamine synthetase

**Fig 3:**
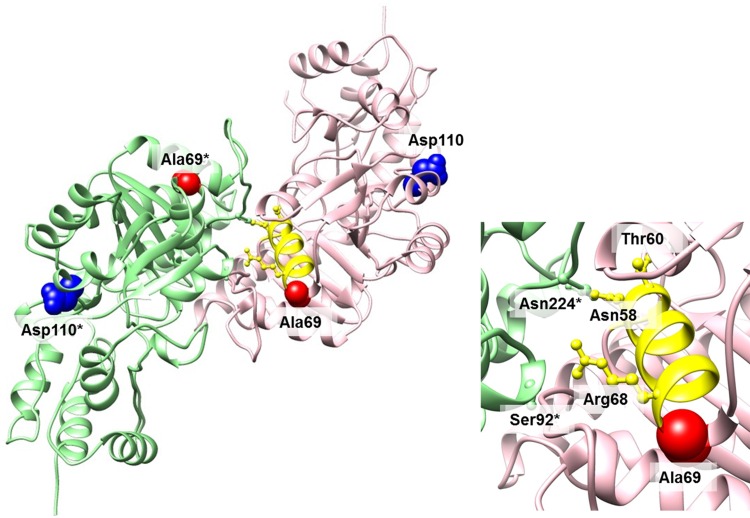
Structural model of FTL_0601 with two mutation positions Ala69, and Asp110 labeled. Left plot: a ribbon representation of two subunits forming homodimer with mutation positions Ala69 and Asp110 shown as spheres colored in red and blue respectively (the asterisk indicates residue from the second subunit of the dimer). Asp110 is located on the surface of the protein within conserved helical region outside the interface area. Right plot: close-up of the region showing Ala69 located at the end of the helical segment Asn58-Ala69 which is a part of the interface between subunits. Examples of three residue positions within this helical region are shown as yellow sticks and their functional importance can be described based on annotation of corresponding positions in other homologous aminotransferases. In particular: two residues Asn58 and Arg68 from both ends of the helix contribute to the interface formation by interacting with Asn224* and Ser92* respectively, the residues Thr60 and Asn224* are both highly conserved and are involved in stabilizing interaction between ligand and protein [21–24]. Ala69 is located on the edge of the interface in close proximity of these residues.

A total of two mutations were identified in this gene from two isolates. The mutation at position 589,311 (amino acid position 110) is a synonymous mutation while the mutation at 589,187 is a non-synonymous mutation. The wild-type triplet encodes Ala at position 69 in the corresponding protein. The mutation found results in a Glu at this position. One of the closest structural templates for modeling of FTL_0601 is a crystal structure of DesI from *Streptomyces venezuelae* (PDB chain: 2po3_A). The level of sequence identity between FTL_0601 and 2po3_A is 29%. Another identified structural template with a similar level of sequence identity is GDP-perosamine synthase from *Caulobacter crescentus* (PDB chain: 3dr4_C). Both templates belong to the same aspartate aminotransferase superfamily. The functional units of enzymes in this group are typically formed as homodimers with an extensive subunit/subunit interface [21]. Construction of a structural model of FTL_0601 and comparative analysis with these two proteins suggests that the corresponding interface in FTL_0601 is formed by the following segments: Lys8-Asn32, N58-Arg68, F83-Asn93, Ile188-E189, F207-Ile212, and Ile218-F230. In Fig 3 examples of critical residues (colored as yellow sticks) found within these segments were shown: interacting residues Arg68 and Ser92, and highly conserved residues Thr60 and Asn224 which are involved in stabilizing interaction between ligand and protein [22]. The detected mutation associated with Cipro resistance is located at Ala69; in immediate proximity to these critical residues (Fig 3). Recently published studies [23, 24]on structural analysis of active sites of different aminotransferases suggests that the observed differences in residues in close proximity to functionally critical residues may be crucial for enzyme function, substrate binding and specificity. Results from the StralSV analysis indicate that the Ala69 position can absorb substitutions with different types of amino acids without significant conformational change of the backbone structure (terminal part of the α-helix). The list of observed diversity of amino acids at the corresponding position in homologous proteins is provided in the Table 2.

#### Mutations in FTL_1547 encoding DNA gyrase B

**Fig 4:**
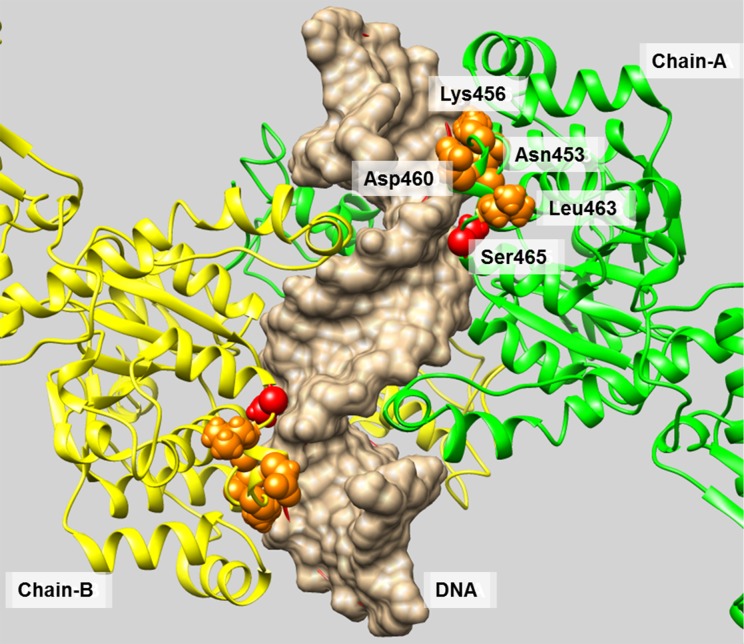
Structural model of FTL_1547 in its dimeric conformation (chains A and B) complexed with DNA. A mutation position Ser465 is colored in red. Ser465 is located next to positions 463, 460, 453 and 456 (colored in orange) that correspond to 471, 468, 464 and 461 from the X-ray structure (PDB chain 4i3h_A) described in [32] as critical functional positions located in the helical region of the C-gate facilitating DNA (T-segment) release.

FTL_1547 is a type II DNA topoisomerase enzyme that catalyzes topological rearrangement of double-stranded DNA by generating a transient double-stranded break in one DNA duplex (the ‘G’ or gate segment) and passing another duplex (the ‘T’ or transported segment) through the break before resealing it. It is a known target for Cipro resistance. A SNP at location 1,477,418 was identified by microarray analysis and Illumina sequencing. Only one isolate 18:5:2 from the 11 Cipro resistant isolates contained this mutation. Sanger sequencing results showed that none of the first round isolates tested contained this mutation but the majority of the second round Cipro resistant isolates had this mutation. This suggests that the mutation occurred during the second round of the selection at higher Cipro concentrations. The mutation results in an amino acid change from Ser to Tyr and is located next to positions 463, 460, 453 and 456. These positions correspond to 471, 468, 464 and 461 from the X-ray structure of topoisomerase from *Streptococcus pneumoniae* (PDB chain: 4i3h_A) and are described in [25] as critical functional positions located in the helical region of the C-gate facilitating DNA (T-segment) release (Fig 4). A second mutation was identified by Illumina sequencing at position 1,477,419, but this mutation was not detected in the analysis of the microarray data.

### Multi drug resistance analysis of Cipro resistant FT LVS mutants

*F*. *tularensis* Ciprofloxacin resistant isolates were resistant to several of the antibiotics while the wild type strain was only resistant to erythromycin. Nearly all of the 148 tested round three mutants were found to grow in elevated concentrations of ampicillin, erythromycin, nalidixic acid, and vancomycin compared to the sensitive isolate from which they were derived. In this group, only the specific MIC values for ampicillin were tested. The wild type strain had a MIC of 3 μg/mL while the tested mutants had a range of 1.5 μg/mL to > 256 μg/mL. Thirty-one of the 148 isolates were resistant to amoxicillin with MICs from 1.5 μg/mL to > 256 μg/mL, the wild type was resistant to 2 μg/mL. Sixty-seven mutants also showed some level of resistance to carbenicillin. The MIC values for the 11 isolates subjected to microarray analysis and Illumina sequencing are listed in Table 3. The drug sensitivity or resistance results from all antibiotics tested on all third round mutants are shown in S5 Table.

**Table 3:** Multiple drug resistance testing of *F*. *tularensis* LVS Ciprofloxacin resistant clones. Units are in μg/mL.

	AmC	Am10	CB100	CIP5	D30	E15	GM10	NA30	RA5	S10	VA30	AmC	Am10	CIP5	TZ	D30	E15	RA5
**F. tularensis LVS Wild Type**	S	S	S	S	S	**R**	S	S	S	S	S	2	3	0.023	U	U	>256	U
C1:1:5	**R**	**R**	S	**R**	S	**R**	S	**R**	S	S	**R**	32	>256	>32	U	U	U	U
C5:8:3	**M**	**R**	**M**	**R**	S	**R**	S	**R**	S	S	S	6	48	>32	U	U	U	U
C8:7:2	**R**	**R**	S	**R**	S	**R**	S	**R**	S	S	**R**	12	>256	24	U	U	U	U
C11:4:3	**M**	**R**	**M**	**R**	S	**R**	S	**R**	S	S	S	8	64	32	U	U	U	U
C12:3:4	**R**	**R**	S	**R**	S	**R**	S	**R**	S	S	**R**	8	>256	>32	U	U	U	U
C14:6:5	**R**	**R**	**M**	**R**	S	**R**	S	**R**	S	S	**R**	8	>256	32	U	U	U	U
C15:6:5	**R**	**R**	**R**	**R**	S	**R**	S	**R**	S	S	**R**	>256	>256	U	U	U	U	U
C16:10:2	**R**	**R**	S	**R**	S	**R**	S	**R**	S	S	**R**	>256	>256	U	U	U	>256	U
C18:5:2	S	**R**	S	**M**	S	**R**	S	**M**	S	S	S	2	48	U	U	U	U	U
C21:8:2	**R**	**R**	**R**	**R**	S	**R**	S	**R**	S	S	**R**	24	>256	U	U	U	U	U
C23:2:4	**M**	**M**	S	**R**	S	**R**	S	**M**	S	S	**R**	U	U	U	U	U	U	U

AmC = Amoxicillin, Am10 = Ampicillin, Cb100 = Carbenicillin, CIP5 = Ciprofloxacin, E15 = Erythromycin, GM10 = Gentamicin, D30 = Doxycycline, VA30 = Vancomycin, NA30 = Nalidixic Acid, RA5 = Rifampin, S10 = Streptomycin

S = Susceptible, I = Increased Sensitivity, M = Moderate, R = Resistant, U = Untested

## Discussion

In this study, we performed the first reported genome-wide study to identify mutations that are associated with Cipro resistance in *F*. *tularensis* LVS. More than 200 first-, second- and third-round mutants were selected for Cipro resistance. A combination of high density tiling microarray, Illumina and Sanger sequencing was used to identify and confirm mutations in these isolates. Structural modeling of the proteins encoded by genes containing these mutations was performed to analyze the structural impacts of these mutations.

The LVS strain (type B) was selected for the Cipro resistance studies because it is fully attenuated and can therefore safely be used for such studies. An analysis of the small number of fully virulent strains that have already demonstrated resistance to Cipro would likely show only the most likely one-step mutations that confer resistance to clinically relevant concentrations of this antibiotic. By doing an extensive, multi-step selection, a broader range of single mutations that could confer resistance to this drug could be identified. Moreover, the use of a multi-step process would select for combinations of mutations that jointly confer resistance to higher drug doses. These combinations would be unlikely to occur naturally in the absence of continued selective pressure by exposure to increased Cipro concentrations, and would be potential signatures of an intentional selection program.

The known mechanism of action for fluoroquinolones is inhibition of certain bacterial topoisomerase enzymes, DNA gyrase and topoisomerase IV. Alternations in target enzymes appear to be the most dominant factors in expression of resistance to quinolones [26]. Prior studies have demonstrated that species naturally bearing a serine residue at position 83 of gyrA are usually susceptible to fluoroquinolones, whereas the presence of an alanine at this critical position corresponds to natural resistance to these antibiotics [9, 27]. An amino acid change from aspartic acid to asparagine at position 87 of gyrA has been reported recently in a Cipro-resistant *B*. *bacilliformis* strain [9, 28]. A recent study by Sutera *et al*. also observed mutations at gyrA-83 and gyrA-87 in Cipro resistant *F*. *tularensis* LVS mutants [29]. Mutations in topoisomerase genes have also been identified in some virulent strains of *F*. *tularensis*. A mutation in *gyr*A (C524->T) was identified in a Cipro-resistant clinical isolate of the virulent *F*. *tularensis* holarctica strain URFT1 using a pyrosequencing technique [30]. In another study, pyrosequencing of the *gyrA*, *parC*, *gyrB* and *parE* genes of a Cipro resistant isolate of *F*. *tularensis* Schu S4 identified two mutations in *gyrA*, G248->T and G259->T, as well as a 5-bp deletion in *parE* [31]. The *gyrA* mutations G248->T and G259->T were also observed in some of the Cipro-resistant LVS isolates in our own study, indicating that similar resistance mechanisms may apply to both type A strains such as Schu S4 and type B strains such as LVS. The four topoisomerase genes have 99% sequence identity between the LVS and Schu S4 genomes, and are 100% identical in the regions where we observed mutations in resistant LVS isolates.

Our study showed that *gyrA* mutations at AA positions 83 and 87 can occur in the same Cipro resistant *F*. *tularensis* LVS isolate and that the combination of two mutations results in a resistance to a higher Cipro concentration than is conferred by either mutation alone. In addition, the two mutations were present in almost all the first and second round mutants, in addition to the third round mutants. We also identified mutations in *gyrB* (1394G->T; S464Y) which encodes the subunit B of DNA gyrase and DNA topoisomerase IV subunit A, both known Cipro targets. These mutations were also identified by a recent study [29].

In addition to the known and novel mutations in *gyrA* and *gyrB*, we identified variants in a number of other genes that have not previously been reported to be involved in Cipro resistance in this species. These include mutations in a hypothetical protein (FTL_0439), an asparagine synthase (FTL_0600), a sugar transamine/perosamine synthetase (FTL_0601), and a few others.

Multiple SNPs were identified in FTL_0439, which encodes a hypothetical protein. Such mutations have not been reported previously. FTL_0439 has been reported to be the remnant of two ORFs that have been fused in the LVS genome by a 1.5 kB deletion [32]. The component ORFs are homologous to Schu S4 loci FTT_0918 and FTT_0919, which also have unknown function. This deletion and gene fusion is unique to the LVS genome, suggesting that it is partly responsible for the attenuated virulence of this strain. Deletion of the FTT_0918 locus from the *F*. *tularensis* Schu S4 genome produces a strain with reduced virulence in mice [33]; this provides further evidence for the role of the FTL_0439 fusion in attenuation of LVS. Since the protein product is unique to LVS and has unknown function, it is unclear whether mutations homologous to the ones we observed in Cipro-resistant LVS isolates would have similar effects in other strains of *F*. *tularensis*. Structural homology studies suggest that FTL_0439 encodes an outer membrane protein that could be involved in ion transport or possibly transport of Cipro into or out of the cell; however, this protein only has 15% homology to any known structural templates, so our conjectures about its function remain highly speculative. For the same reason, it is unclear whether there is any connection between the role of FTL_0439 in the reduced pathogenicity of LVS and the effect of mutations in this gene on Cipro susceptibility.

Our studies showed that both high density microarray and Illumina sequencing are capable of identifying rare mutations and can be used to compare genomic differences between different bacterial isolates of the same species and between sensitive and antibiotic resistant isolates. It is important that at least 85% overlap of tiling probes are needed in array design to identify the minor genomic differences. A previous design on a different bacterium using a 50% overlap did not provide high confidence SNP calling (unpublished data). The use of a sensitive analysis algorithm was also needed to predict the genomic differences between a reference strain and an antibiotic resistance isolate derived from the same strain.

Comparison of microarray and Illumina sequencing data from the different isolates showed that 23 mutations were identified by both array and Illumina sequencing, while 8 mutations were identified by Illumina sequencing only. Microarray analyses detected more apparent mutations than were identified by Illumina sequencing. These presumed false positives could be due to the errors caused by hybridization efficiency. It is also possible that the >85% tiling overlap was still not sensitive enough to accurately detect all mutations.

Further studies to verify the role of the identified mutations are necessary. One such approach is to revert a mutation thought to confer Cipro resistance back to wild-type then measure changes in resistance. We have performed such studies with *B*. *anthracis* Sterne and shown that replacement of the mutant *gyrA* gene with a wild-type *gyrA* reduces the Cipro MIC back to that found in the Cipro-sensitive isolate (unpublished data). This approach can be used to verify the genes that confer mechanisms of resistance in *F*. *tularensis* by generating deletion mutants using methods developed by Horzempa *et al*. [34] and LoVullo et al. [35].

This study provides insights into possible molecular mechanisms behind resistance to antibiotics commonly used to treat infections caused by *F*. *tularensis* and other related Gram negative bacteria. Once these putative mechanisms have been shown to contribute to antibiotic resistance, such information can be used to develop assays that can rapidly detect these molecular signatures without growth of the microbe. They should also provide insights into previously unknown mechanisms of antibiotic resistance and how these might be defeated therapeutically.

It is not surprising that selection for resistance to Cipro results in co-resistance to nalidixic acid, another quinolone that targets the same proteins as ciprofloxacin. However, it is more difficult to understand why selection for resistance to Cipro would result in isolates with mutations in genes that target unrelated antibiotics. Amoxicillin, ampicillin and carbenicillin are beta-lactam antibiotics in the penicillin family. Clavulanic acid is a beta-lactamase inhibitor. Amoxicillin/clavulanic acid resistance has been linked to an increase in the expression of beta-lactamase or inhibitor-resistant TEM enzymes [36, 37]. Ampicillin resistance is linked to TEM 1 beta-lactamase in enterobacteriaceae [38, 39] or mutations in penicillin binding protein 4 [39, 40]. Carbenicillin can be used in place of ampicillin. Studies in *Staphylococcus aureus* have demonstrated that exposure to sub-lethal Ciprofloxacin concentrations results in genomic alterations linked to rifampin resistance [41]. Perhaps growth of first and second round Cipro-resistant populations in higher Cipro concentrations results in introduction of such mutations at a higher frequency in those isolates that manifest a mutation pattern that allows rapid growth in the Cipro concentration used for selection.

## Supporting Information

S1 Fig The distributions of log intensity ratios for probes overlapping known sequence variations between the reference LVS strain and the SchuS4 strain hybridized to a typical array. The probes are grouped by the reference triplet centered at the SNP locus. As expected, SNPs affecting a triplet with a central G or C base have a stronger effect on average than those replacing an A or a T.
(PDF)

S2 Fig A typical profile of intensity change vs SNP position for the same SchuS4 vs LVS array. Each column in this plot represents the distribution of log intensity ratios between the Cy3 (LVS) and Cy5 (SchuS4) channels, for probes overlapping a SchuS4 variation at a given position in the probe; the central bar represents the range from the 25^th^ to the 75^th^ percentiles.
(PDF)

S1 Table Avirulent *F*. *tularensis* LVS Cipro resistant isolate minimal inhibitory concentration (MIC) summary. All MIC values are in μg/ml.
(DOCX)

S2 Table List of genes with mutations identified from more than two *F*. *tularensis* Cipro resistant clones by microarray. The genes are listed under gene locus order.
(DOCX)

S3 Table SNPs identified from *F*. *tularensis* Cipro resistant clones on both strands. The location of the SNP, the number of reads and the percentage of reads containing the SNPs are included.
(XLSX)

S4 Table SNPs that were detected from *F*. *tularensis* Cipro resistant clones by sequencing, but not by microarray. The lists of identified amino acid diversities at the given mutation points observed in the corresponding positions in homologous proteins are provided in the “Amino acid change” column.
(DOCX)

S5 Table *F*. *tularensis* LVS Cipro resistant isolate multiple drug resistance testing results. Results from all third round mutants tested against the various antibiotics are shown.
(DOCX)
