# Supplementary figures and images for: Identification of Genome-Wide Mutations in Ciprofloxacin-Resistant F. tularensis LVS Using Whole Genome Tiling Arrays and Next Generation Sequencing
Source: PLoS One. 2016 Sep 26;11(9):e0163458. doi: 10.1371/journal.pone.0163458 (PMC5036845; doi:10.1371/journal.pone.0163458)

Normalized Log Ratio

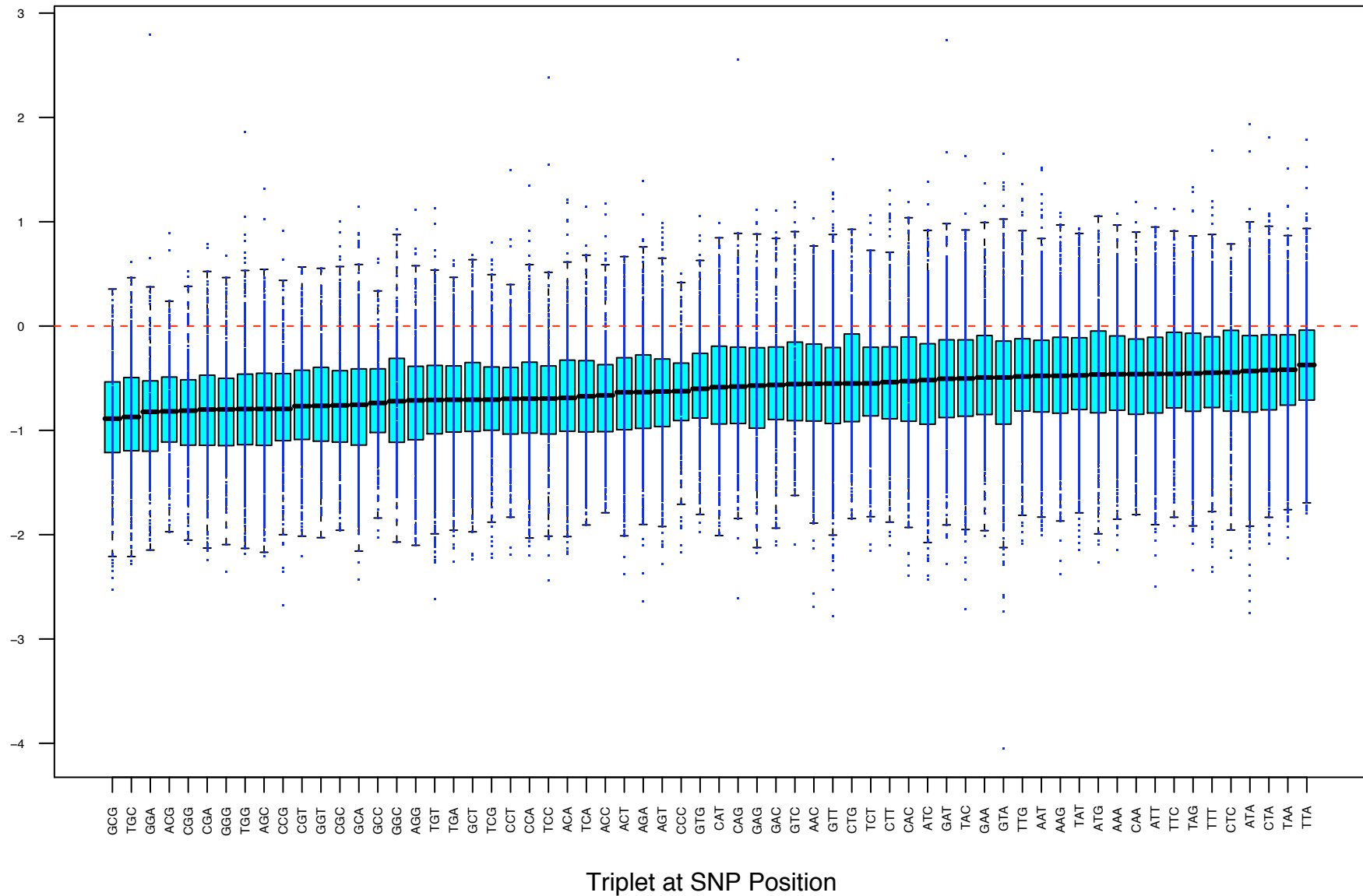

Supplement: S1 Fig — The probes are grouped by the reference triplet centered at the SNP locus. As expected, SNPs affecting a triplet with a central G or C base have a stronger effect on average than those replacing an A or a T. (PDF) [file pone.0163458.s001.pdf]

Normalized Log Ratio

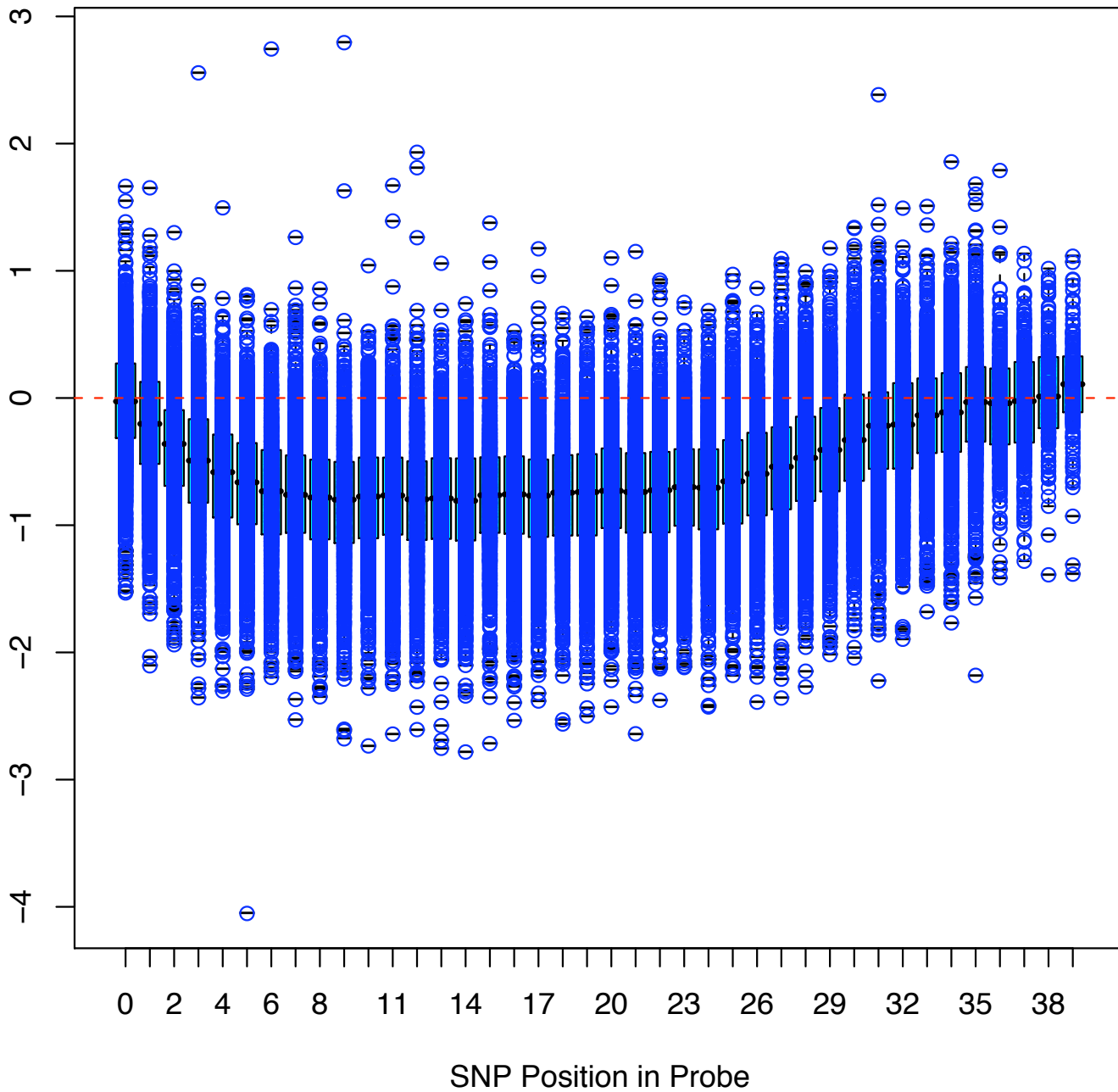

Supplement: S2 Fig — Each column in this plot represents the distribution of log intensity ratios between the Cy3 (LVS) and Cy5 (SchuS4) channels, for probes overlapping a SchuS4 variation at a given position in the probe; the central bar represents the range from the 25th to the 75th percentiles. (PDF) [file pone.0163458.s002.pdf]
