# Supplementary material for: Identification of Genome-Wide Mutations in Ciprofloxacin-Resistant F. tularensis LVS Using Whole Genome Tiling Arrays and Next Generation Sequencing
Source: PLoS One. 2016 Sep 26;11(9):e0163458. doi: 10.1371/journal.pone.0163458 (PMC5036845; doi:10.1371/journal.pone.0163458)
Supplement: S1 Table — All MIC values are in μg/ml. (DOCX) [file pone.0163458.s003.docx]

S1 Table. **Avirulent *F. tularensis* LVS Cipro resistant isolate minimal inhibitory concentration (MIC) summary.** All MIC values are in µg/ml.

| **Avirulent *F. tularensis* LVS – wild-type ciprofloxacin MIC (Etest) = 0.023 µg/ml** | | | | | |
| --- | --- | --- | --- | --- | --- |
| **Round 1** | | **Round 2** | | **Round 3** | |
| **Name** | **MIC** | **Name** | **MIC** | **Name** | **MIC** |
| M1 | 0.75 | M2:1 | 2.0 | M1:1:2 | 12 |
| M2 | 0.25 | M2:4 | 2.0 | M1:1:3 | 12 |
| M3 | 0.38 | M2:5 | 1.5 | M1:1:5 | > 32 |
| M4 | 0.38 | M2:6 | 2.0 | M5:1:2 | 8 |
| M5 | 0.38 | M2:7 | 2.0 | M5:1:3 | 6 |
| M6 | 0.5 | M2:8 | 1.0 | M5:1:5 | 12 |
| M7 | 1.0 | M2:9 | 1.5 | M5:1:6 | 12 |
| M8 | 0.75 | M2:10 | 1.5 | M5:2:2 | 32 |
| M9 | 0.5 | M3:1 | 1.0 | M5:2:3 | 12 |
| M10 | 0.5 | M3:3 | 1.5 | M5:2:4 | 24 |
| M11 | 0.38 | M3:5 | 1.0 | M5:2:5 | 16 |
| M12 | 0.5 | M3:6 | 1.5 | M5:2:6 | 12 |
| M13 | 0.5 | M3:7 | 1. 0 | M5:3:1 | 32 |
| M14 | 0.5 | M3:8 | 2. 0 | M5:3:2 | 32 |
| M15 | 0.25 | M3:9 | 1.5 | M5:3:3 | 12 |
| M16 | 0.38 | M3:10 | 2. 0 | M5:3:4 | 32 |
| M17 | 0.5 | M5:1 | 0.75 | M5:3:5 | > 32 |
| M18 | 0.25 | M5:2 | 1.5 | M5:3:6 | 24 |
| M19 | 0.5 | M5:3 | 1.5 | M5:4:1 | > 32 |
| M20 | 0.38 | M5:4 | 2 | M5:4:2 | > 32 |
| M21 | 0.5 | M5:5 | 1.5 | M5:4:3 | 8 |
| M22 | 0.75 | M5:6 | 1.5 | M5:4:4 | > 32 |
| M23 | 0.25 | M5:7 | 1.5 | M5:4:5 | 32 |
| M24 | 0.75 | M5:8 | 2 | M5:4:6 | 12 |
| M25 | 1.0 | M5:9 | 1 | M5:5:2 | 12 |
| M26 | 0.75 | M5:10 | 1 | M5:5:3 | 12 |
| M27 | 1.0 | M8:2 | 4 | M5:5:5 | 12 |
| M28 | 0.5 | M8:7 | 3 | M5:6:2 | 16 |
|  |  | M11:1 | 1.5 | M5:6:3 | 12 |
|  |  | M11:3 | 3 | M5:6:5 | 12 |
|  |  | M11:4 | 1.5 | M5:6:6 | 24 |
|  |  | M11:7 | 2 | M5:7:1 | > 32 |
|  |  | M11:8 | 1.5 | M5:7:3 | 24 |
|  |  | M11:10 | 1.5 | M5:7:5 | 12 |
|  |  | M12:2 | 4 | M5:7:6 | 8 |
|  |  | M12:3 | 0.75 | M5:8:1 | 6 |
|  |  | M12:6 | 2 | M5:8:2 | 12 |
|  |  | M12:8 | 4 | M5:8:3 | > 32 |
|  |  | M13:10 | 0.5 | M5:8:4 | 16 |
|  |  | M14:6 | 4 | M5:8:5 | 32 |
|  |  | M14:7 | 2 | M5:8:6 | > 32 |
|  |  | M14:8 | 2 | M5:9:2 | 24 |
|  |  | M14:10 | 1.5 | M5:9:5 | 32 |
|  |  | M14:6 | 4 | M5:10:2 | 8 |
|  |  | M14:7 | 2 | M5:10:5 | 8 |
|  |  | M14:8 | 2 | M8:2:1 | 8 |
|  |  | M14:10 | 1.5 | M8:2:3 | 12 |
|  |  | M15:1 | 3 | M8:2:4 | 12 |
|  |  | M15:2 | 8 | M8:2:5 | 12 |
|  |  | M15:3 | 1.5 | M8:2:6 | 16 |
|  |  | M15:4 | 3 | M8:7:2 | 24 |
|  |  | M15:6 | 1 | M8:7:3 | 8 |
|  |  | M15:9 | 3 | M8:7:5 | 12 |
|  |  | M16:1 | 3 | M8:7:6 | 32 |
|  |  | M16:2 | 3 | M11:3:1 | > 32 |
|  |  | M16:3 | 2 | M11:3:2 | > 32 |
|  |  | M16:4 | 3 | M11:3:3 | > 32 |
|  |  | M16:6 | 4 | M11:3:4 | > 32 |
|  |  | M16:7 | 16 | M11:3:5 | > 32 |
|  |  | M16:8 | 4 | M11:3:6 | > 32 |
|  |  | M16:9 | 3 | M11:4:3 | 32 |
|  |  | M16:10 | 3 | M11:7:2 | 32 |
|  |  | M17:8 | 3 | M11:10:1 | 8 |
|  |  | M17:9 | 3 | M11:10:2 | 12 |
|  |  | M17:10 | 4 | M11:10:3 | 6 |
|  |  | M18:1 | 3 | M11:10:4 | 8 |
|  |  | M18:2 | 4 | M11:10:5 | 12 |
|  |  | M18:3 | 4 | M12:2:2 | > 32 |
|  |  | M18:4 | 4 | M12:2:3 | > 32 |
|  |  | M18:5 | 6 | M12:3:1 | > 32 |
|  |  | M18:6 | 4 | M12:3:2 | > 32 |
|  |  | M18:7 | 6 | M12:3:3 | > 32 |
|  |  | M18:8 | 3 | M12:3:4 | > 32 |
|  |  | M18:9 | 3 | M12:3:5 | > 32 |
|  |  | M18:10 | 4 | M12:3:6 | > 32 |
|  |  | M19:10 | 4 | M12:8:5 | > 32 |
|  |  | M20:1 | 3 | M14:6:1 | 32 |
|  |  | M20:3 | 1.5 | M14:6:5 | 32 |
|  |  | M20:4 | 2 | M14:7:4 | 32 |
|  |  | M20:5 | 2 | M14:6:1 | 16 |
|  |  | M20:6 | 2 | M14:10:1 | 32 |
|  |  | M20:7 | 1.5 | M14:10:4 | 12 |
|  |  | M20:8 | 2 | M15:4:6 | > 32 |
|  |  | M20:9 | 2 | M15:6:2 | > 32 |
|  |  | M20:10 | 1.5 | M15:6:3 | > 32 |
|  |  | M21:4 | 1.5 | M15:6:4 | > 32 |
|  |  | M21:7 | 1.5 | M15:6:5 | 24 |
|  |  | M21:8 | 1 | M16:3:2 | 24 |
|  |  | M22:1 | > 32 | M16:3:2 | 24 |
|  |  | M22:7 | 2 | M16:3:6 | 24 |
|  |  | M23:2 | 1.5 | M16:4:1 | > 32 |
|  |  | M23:3 | 2 | M16:7:2 | > 32 |
|  |  | M23:4 | 1.5 | M16:7:3 | > 32 |
|  |  | M23:5 | 3 | M16:7:4 | > 32 |
|  |  | M23:6 | 3 | M16:7:5 | > 32 |
|  |  | M23:7 | 3 | M16:7:6 | > 32 |
|  |  | M23:8 | 1 | M16:8:3 | > 32 |
|  |  | M23:9 | 2 | M16:8:5 | > 32 |
|  |  |  |  | M16:8:6 | > 32 |
|  |  |  |  | M16:9:1 | > 32 |
|  |  |  |  | M16:9:2 | > 32 |
|  |  |  |  | M16:10:2 | > 32 |
|  |  |  |  | M16:10:3 | > 32 |
|  |  |  |  | M16:10:4 | > 32 |
|  |  |  |  | M16:10:5 | > 32 |
|  |  |  |  | M16:10:6 | > 32 |
|  |  |  |  | M17:8:6 | > 32 |
|  |  |  |  | M17:9:1 | 24 |
|  |  |  |  | M17:9:6 | 24 |
|  |  |  |  | M17:10:3 | > 32 |
|  |  |  |  | M18:1:1 | > 32 |
|  |  |  |  | M18:3:3 | > 32 |
|  |  |  |  | M18:5:1 | > 32 |
|  |  |  |  | M18:5:2 | > 32 |
|  |  |  |  | M18:5:3 | > 32 |
|  |  |  |  | M18:6:1 | > 32 |
|  |  |  |  | M18:7:1 | 6 |
|  |  |  |  | M23:2:1 | 32 |
|  |  |  |  | M23:2:2 | > 32 |
|  |  |  |  | M23:2:3 | > 32 |
|  |  |  |  | M23:2:4 | 32 |
|  |  |  |  | M23:4:2 | > 32 |
