# Supplementary material for: Identification of Genome-Wide Mutations in Ciprofloxacin-Resistant F. tularensis LVS Using Whole Genome Tiling Arrays and Next Generation Sequencing
Source: PLoS One. 2016 Sep 26;11(9):e0163458. doi: 10.1371/journal.pone.0163458 (PMC5036845; doi:10.1371/journal.pone.0163458)
Supplement: S2 Table — The genes are listed under gene locus order. (DOCX) [file pone.0163458.s004.docx]

S2 Table. **List of genes with mutations identified from more than two *F. tularensis*** **Cipro resistant clones by microarray**. The genes are listed under gene locus order.

| **Gene annotation** | **Locus** | **Clone #** |
| --- | --- | --- |
| conserved hypothetical membrane protein\|\|FTL_0057\| | 57386 | 23:2:4 |
|  | 57511 | 14:6:5 |
|  | 57832 | 15:6:5 |
| conserved hypothetical membrane protein\|\|FTL_0206\| | 205911 | 15:6:5 |
|  | 205914 | 21:8:2 |
| uridylate kinase\|\|FTL_0226\| | 226927 | 14:6:5 |
|  | 227097 | 15:6:5 |
| phosphatidate cytidylyltransferase\|\|FTL_0229\| | 229262 | 14:6:5 |
|  | 229460 | 15:6:5 |
|  | 229480 | 15:6:5 |
| elongation factor G (EF-G)\|\|FTL_0234\| | 231982 | 15:6:5 |
|  | 232175 | 5:8:3 |
|  | 232217 | 15:6:5 |
|  | 232287 | 15:6:5 |
|  | 232370 | 15:6:5 |
|  | 232394 | 15:6:5 |
|  | 232452 | 15:6:5 |
|  | 232538 | 15:6:5 |
|  | 232721 | 15:6:5 |
|  | 232761 | 15:6:5 |
|  | 232774 | 14:6:5 |
| intergenic | 351151 | 15:6:5, 21:8:2 |
| hypothetical protein\|\|FTL_0439\| | 406472 | 15:6:5 |
|  | 407047 | 21:8:2 |
|  | 407701 | 1:1:5, 11:4:3 |
|  | 407703 | 12:3:4 |
|  | 407706 | 18:5:2 |
|  | 407708 | 23:2:4 |
| ABC transporter, membrane protein\|\|FTL_0516\| | 498547 | 15:6:5, 21:8:2 |
| DNA gyrase, subunit A\|\|FTL_0533\| | 514142 | 12:3:4, 14:6:5, 1:1:5, 8:7:2 |
|  | 514145 | 21:8:2 |
|  | 514148 | 5:8:3 |
|  | 514150 | 11:4:3 |
|  | 514153 | 16:10:2 |
|  | 514157 | 23:2:4 |
|  | 514159 | 15:6:5 |
|  | 514160 | 18:5:2 |
| asparagine synthase\|\|FTL_0600\| | 588302 | 15:6:5 |
|  | 588914 | 11:4:3 |
| sugar transamine/perosamine synthetase\|\|FTL_0601\| | 589192 | 8:7:2 |
|  | 589315 | 15:6:5 |
|  | 589652 | 15:6:5 |
| conserved hypothetical protein, pseudogene\|\|FTL_0824\| | 806163 | 11:4:3, 5:8:3 |
| phosphogylcerate kinase\|\|FTL_1147\| | 1090255 | 16:10:2 |
|  | 1090261 | 18:5:2 |
| virulence factor MviN\|\|FTL_1305\| | 1242645 | 5:8:3, 11:4:3 |
| intergenic preceding outer membrane associated protein\|\|FTL_1328\| | 1264103 | 21:8:2 |
|  | 1264170 | 15:6:5 |
