# Supplementary material for: Identification of Genome-Wide Mutations in Ciprofloxacin-Resistant F. tularensis LVS Using Whole Genome Tiling Arrays and Next Generation Sequencing
Source: PLoS One. 2016 Sep 26;11(9):e0163458. doi: 10.1371/journal.pone.0163458 (PMC5036845; doi:10.1371/journal.pone.0163458)
Supplement: S4 Table — The lists of identified amino acid diversities at the given mutation points observed in the corresponding positions in homologous proteins are provided in the “Amino acid change” column. (DOCX) [file pone.0163458.s006.docx]

S4 Table. **SNPs that were detected from *F. tularensis* Cipro resistant clones by sequencing, but not by microarray**. The lists of identified amino acid diversities at the given mutation points observed in the corresponding positions in homologous proteins are provided in the “Amino acid change” column.

| **Gene annotation** | **Mutation and codon context** | **Amino acid change**  **(AA diversity in homologous proteins** **in order from most to least frequent)** | **Cipro resistant isolates containing this mutation** | **Reference Genome Position*** | **% of reads contain this mutation** |
| --- | --- | --- | --- | --- | --- |
| Hypothetical protein [FTL_0439]  region(406452..408107) | G−>T  ATTAGGAGG | G-167−>stop  (G,Y,E,V,N,D) | 16:10:2 | 406,950 | >90% |
| DNA topoisomerase IV subunit A  [FTL_0462]  region(436126..436134) | C−>A  GCTGCGATG | A-120−>E  (A,S,Y,F,E,P,H) | 5:8:3 | 436,130 | 55% |
| Phosphoglucomutase, [FTL_0484]  region(465981..467615) | G−>T  AATGGTATA | G-88−>V  (G,A,S,T,V,I) | 14:6:5 | 466,243 | 66% |
| Hypothetical protein [FTL_0544]  complement(527080..527889) | G−>T  TCTTGTTCT | Q-93−>K  (R,E,T,V,K,H,D,Q,Y) | 23:2:4 | 527,613 | >90% |
| dTDP-glucose 4,6-dehydratase  [FTL_0592]  region(578226..579962) | 7 T insertion | - | 23:2:4 | 578,478-84 | >90% |
| RNA polymerase factor sigma-32  [FTL_0851]  region(832559..833437) | A−>T  GATAATTTC | N-227−>I  (N,Q,H,R,S,T,G,L,A,D,V,K,E,I) | 23:2:4 | 833,238 | >90% |
| Hypothetical protein [FTL_0872]  region(851235..851582) | G−>A  GCTCGTTTT | S-41−>S  (S,V,L,R,T,A) | 23:2:4 | 851,357 | >90% |
| DNA gyrase subunit B [FTL_1547]  complement(1476400..1478811) | A−>C  TGAGAACCA | S-465−>A  (S,N,A,Y,H,F) | 23:2:4 | 1,477,419 | >90% |
| 50S ribosomal protein L11 [FTL_1748]  complement(1683387..1683821) | A−>C  TAATAGCTT | A-8−>A  (G,A,K,T,Q) | 1:1:5 | 1,683,798 | 64% |
