# Supplementary material for: Identification of Genome-Wide Mutations in Ciprofloxacin-Resistant F. tularensis LVS Using Whole Genome Tiling Arrays and Next Generation Sequencing
Source: PLoS One. 2016 Sep 26;11(9):e0163458. doi: 10.1371/journal.pone.0163458 (PMC5036845; doi:10.1371/journal.pone.0163458)
Supplement: S5 Table — Results from all third round mutants tested against the various antibiotics are shown. (DOCX) [file pone.0163458.s007.docx]

S5 Table. ***F. tularensis* LVS Cipro resistant isolate multiple drug resistance testing** **results.** Results from all third round mutants tested against the various antibiotics are shown.

| **Francisella tularensis LVS Ciprofloxacin Resistant Isolates** | | | | | | | | |  |  |  |  |  |  |  |  |  |  |
| --- | --- | --- | --- | --- | --- | --- | --- | --- | --- | --- | --- | --- | --- | --- | --- | --- | --- | --- |
|  |  |  |  |  |  |  |  |  |  |  |  |  |  |  |  |  |  |  |
|  | **AmC** | **Am10** | **CB100** | **CIP5** | **D30** | **E15** | **GM10** | **NA30** | **RA5** | **S10** | **VA30** | **AmC** | **Am10** | **CIP5** | **TZ** | **D30** | **E15** | **RA5** |
| **F. tularensis LVS Wild Type** | S | S | S | S | S | **R** | S | S | S | S | S | 2 | 3 | 0.023 | U | U | >256 | U |
| C1:1:2 | S | **R** | S | **R** | S | **R** | S | **M** | S | S | **R** | 12 | >256 | 12 | U | U | U | U |
| C1:1:3 | **R** | **R** | S | **R** | S | **R** | S | **M** | S | S | **R** | 96 | >256 | 12 | U | U | U | U |
| C1:1:5 | **R** | **R** | S | **R** | S | **R** | S | **R** | S | S | **R** | 32 | >256 | >32 | U | U | U | U |
| C2:1:1 | **R** | **R** | S | **M** | S | **R** | S | **M** | S | S | **M** | 6 | 32 | U | U | U | U | U |
| C2:1:2 | **M** | **R** | S | **M** | S | **R** | S | **M** | S | S | S | 3 | 16 | U | U | U | U | U |
| C2:1:3 | **R** | **R** | **M** | **R** | S | **R** | S | **M** | S | S | **M** | 4 | 6 | U | U | U | U | U |
| C2:1:4 | **M** | **R** | S | **M** | S | **R** | S | **M** | S | S | S | 4 | 96 | U | U | U | U | U |
| C2:1:5 | **M** | **R** | S | **M** | S | **R** | S | **M** | S | S | S | 3 | 12 | U | U | U | U | U |
| C2:1:6 | **R** | **R** | **M** | **M** | S | **R** | S | **M** | S | S | **M** | 6 | 12 | U | U | U | U | U |
| C2:4:1 | **M** | **R** | **M** | **R** | S | **R** | S | **M** | S | S | **M** | 8 | 256 | U | U | U | U | U |
| C2:4:4 | **R** | **R** | S | **R** | S | **R** | S | **R** | S | S | **R** | 16 | >256 | U | U | U | U | U |
| C2:4:6 | **R** | **R** | **R** | **R** | S | **R** | S | **R** | S | S | **R** | 32 | >256 | U | U | U | U | U |
| C2:5:1 | **R** | **R** | **R** | **R** | S | **R** | S | **R** | S | S | **M** | 24 | >256 | U | U | U | U | U |
| C2:5:2 | **M** | **R** | S | **M** | S | **R** | S | **M** | S | S | S | 4 | 8 | U | U | U | U | U |
| C2:5:3 | S | S | S | S | S | **R** | S | **M** | S | S | **M** | 2 | 3 | U | U | U | U | U |
| C2:5:4 | **M** | **R** | S | **M** | S | **R** | S | **M** | S | S | S | 2 | 6 | U | U | U | U | U |
| C2:5:5 | **R** | **R** | **M** | **R** | S | **R** | S | **M** | S | S | **M** | 64 | >256 | U | U | U | U | U |
| C2:5:6 | **R** | **R** | **M** | **R** | S | **R** | S | **M** | S | S | **R** | 48 | >256 | U | U | U | U | U |
| C2:6:6 | **R** | **R** | **M** | **R** | S | **R** | S | **M** | S | S | **M** | 4 | >256 | U | U | U | U | U |
| C3:3:2 | **R** | **R** | **M** | **R** | S | **R** | S | **R** | S | S | **R** | 24 | >256 | U | U | U | U | U |
| C3:3:1 | **R** | **R** | **R** | **R** | S | **R** | S | **R** | S | S | **R** | 16 | >256 | U | U | U | U | U |
| C3:3:3 | **R** | **R** | **M** | **R** | S | **R** | S | **M** | S | S | **R** | 24 | >256 | U | U | U | U | U |
| C3:3:4 | **R** | **R** | **M** | **R** | S | **R** | S | **M** | S | S | **M** | 12 | >256 | U | U | U | U | U |
| C3:3:5 | **R** | **R** | S | **M** | S | **R** | S | **M** | S | S | S | 48 | >256 | U | U | U | U | U |
| C3:3:6 | **R** | **R** | **M** | **M** | S | **R** | S | **M** | S | S | S | U | U | U | U | U | U | U |
| C3:5:1 | **R** | **R** | **M** | **R** | S | **R** | S | **M** | S | S | **R** | 16 | >256 | U | U | U | U | U |
| C3:5:2 | **R** | **R** | **R** | **R** | S | **M** | S | **R** | S | S | **R** | 16 | >256 | U | U | U | U | U |
| C3:5:3 | **R** | **R** | S | **R** | S | **R** | S | **R** | S | S | **R** | 16 | >256 | U | U | U | U | U |
| C3:5:4 | **R** | **R** | **R** | **R** | S | **R** | S | **R** | S | S | **R** | 12 | 96 | U | U | U | U | U |
| C3:5:5 | **R** | **R** | **M** | **R** | S | **R** | S | **M** | S | S | **R** | 8 | 128 | U | U | U | U | U |
| C3:5:6 | **R** | **R** | **M** | **R** | S | **R** | S | **R** | S | S | **R** | 4 | 12 | U | U | U | U | U |
| C3:6:1 | **R** | **R** | **R** | **R** | S | **R** | S | **R** | S | S | **R** | 24 | >256 | U | U | U | U | U |
| C3:6:2 | **R** | **R** | S | **R** | S | **R** | S | **R** | S | S | **R** | 16 | >256 | U | U | U | U | U |
| C3:6:3 | **R** | **R** | **M** | **R** | S | **R** | S | **R** | S | S | **R** | 32 | >256 | U | U | U | U | U |
| C3:6:6 | **R** | S | S | **R** | S | **R** | S | **R** | S | **M** | **R** | 12 | >256 | U | U | U | U | U |
| C5:1:2 | S | **M** | S | **M** | S | **R** | S | **M** | S | S | S | 1.5 | 1.5 | 8 | U | U | U | U |
| C5:1:3 | S | **M** | S | **M** | S | **R** | S | **M** | S | S | S | 3 | 48 | 6 | U | U | U | U |
| C5:1:5 | S | **M** | S | **M** | S | **R** | S | **M** | S | S | S | 2 | 3 | 12 | U | U | U | U |
| C5:1:6 | **M** | **M** | S | **M** | S | **R** | S | **M** | S | S | S | 1.5 | 4 | 12 | U | U | U | U |
| C5:2:2 | **M** | **R** | S | **M** | S | **R** | S | **M** | S | S | S | 1.5 | 3 | 32 | U | U | U | U |
| C5:2:3 | **M** | **M** | S | **M** | S | **R** | S | **M** | S | S | S | 2 | 6 | 12 | U | U | U | U |
| C5:2:4 | S | **M** | S | **M** | S | **R** | S | **M** | S | S | S | 2 | 2 | 24 | U | U | U | U |
| C5:2:5 | S | **M** | S | **M** | S | **R** | S | **M** | S | S | S | 3 | 4 | 16 | U | U | U | U |
| C5:2:6 | S | **M** | S | **M** | S | **R** | S | **M** | S | S | S | 2 | 6 | 12 | U | U | U | U |
| C5:3:1 | S | **M** | S | **M** | S | **R** | S | **M** | S | S | S | 2 | 3 | 32 | U | U | U | U |
| C5:3:2 | S | S | S | **R** | S | **R** | S | **M** | S | S | S | 4 | 8 | 32 | U | U | U | U |
| C5:3:3 | S | **M** | S | **M** | S | **R** | S | **M** | S | S | S | 3 | 4 | 12 | U | U | U | U |
| C5:3:4 | S | S | S | **M** | S | **R** | S | **M** | S | S | S | 2 | 3 | 32 | U | U | U | U |
| C5:3:5 | S | S | S | **M** | S | **R** | S | **M** | S | S | S | 3 | 4 | >32 | U | U | U | U |
| C5:3:6 | S | **M** | S | **M** | S | **R** | S | **M** | S | S | S | 4 | 8 | 24 | U | U | U | U |
| C5:4:1 | **R** | **R** | **M** | **R** | S | **R** | S | **R** | S | S | **R** | 8 | 128 | >32 | U | U | U | U |
| C5:4:2 | **M** | **R** | **M** | **R** | S | **R** | S | **R** | S | S | S | 8 | 48 | >32 | U | U | U | U |
| C5:4:3 | **R** | **R** | **M** | **M** | S | **R** | S | **R** | S | S | S | 8 | 32 | 8 | U | U | U | U |
| C5:4:4 | S | **R** | S | **R** | S | **R** | S | **R** | S | S | S | 48 | >256 | >32 | U | U | U | U |
| C5:4:5 | **R** | **R** | **M** | **R** | S | **R** | S | **R** | S | S | **R** | 12 | >256 | 32 | U | U | U | U |
| C5:4:6 | **M** | **R** | **M** | **R** | S | **R** | S | **M** | S | S | S | 3 | 16 | 12 | U | U | >256 | U |
| C5:5:2 | **R** | **R** | **M** | **R** | S | **R** | S | **M** | S | S | **R** | 4 | 24 | 12 | U | U | U | U |
| C5:5:3 | **M** | S | S | **M** | S | **R** | S | **R** | S | S | S | 4 | 24 | 12 | U | U | U | U |
| C5:5:5 | **R** | **R** | **R** | **R** | S | **R** | S | **R** | S | S | **R** | 4 | 12 | 12 | U | U | U | U |
| C5:6:2 | **R** | **R** | S | **M** | S | **R** | S | **R** | S | S | S | 3 | 16 | 16 | U | U | U | U |
| C5:6:3 | **R** | **R** | **M** | **R** | S | **R** | S | **R** | S | S | **R** | 4 | >256 | 12 | U | U | U | U |
| C5:6:5 | **R** | **R** | **M** | **R** | S | **R** | S | **R** | S | S | **R** | 12 | >256 | 12 | U | U | U | U |
| C5:6:6 | **R** | **R** | **M** | **R** | S | **R** | S | **R** | S | S | **R** | 3 | 6 | 24 | U | U | U | U |
| C5:7:1 | S | S | S | **M** | S | **R** | S | **M** | S | S | **R** | 6 | 64 | >32 | U | U | U | U |
| C5:7:3 | **R** | **R** | **M** | **M** | S | **R** | S | **R** | S | S | **R** | 3 | 24 | 24 | U | U | U | U |
| C5:7:5 | S | S | S | **M** | S | **R** | S | **R** | S | S | S | 8 | >256 | 12 | U | U | U | U |
| C5:7:6 | S | S | S | **M** | S | **R** | S | **M** | S | S | S | 8 | 64 | 8 | U | U | U | U |
| C5:8:1 | **R** | **R** | **M** | **R** | S | **R** | S | **R** | S | S | **R** | 6 | 96 | 6 | U | U | U | U |
| C5:8:2 | **R** | **R** | S | **M** | S | **R** | S | **R** | S | S | S | 4 | 32 | 12 | U | U | U | U |
| C5:8:3 | **M** | **R** | **M** | **R** | S | **R** | S | **R** | S | S | S | 6 | 48 | >32 | U | U | U | U |
| C5:8:4 | **M** | **R** | **M** | **R** | S | **R** | S | **R** | S | S | S | 4 | 32 | 16 | U | U | U | U |
| C5:8:5 | **R** | **R** | S | **M** | S | **R** | S | **M** | S | S | **R** | 6 | >256 | 32 | U | U | U | U |
| C5:8:6 | **M** | **R** | S | **R** | S | **R** | S | **R** | S | S | S | 4 | 128 | >32 | U | U | U | U |
| C5:9:2 | **M** | **R** | S | **R** | S | **R** | S | **M** | S | S | S | 1.5 | 4 | 24 | U | U | U | U |
| C5:9:5 | **R** | **R** | S | **R** | S | **R** | S | **R** | S | S | S | 2 | 4 | 32 | U | U | U | U |
| C5:10:2 | **R** | **R** | **M** | **R** | S | **R** | S | **M** | S | S | **R** | 4 | 8 | 8 | U | U | U | U |
| C5:10:5 | **R** | **R** | S | **M** | S | **R** | S | **M** | S | S | **R** | 8 | 64 | 8 | U | U | U | U |
| C8:2:1 | **R** | **R** | **M** | **R** | S | **R** | S | **R** | S | S | **R** | 24 | >256 | 8 | U | U | U | U |
| C8:2:3 | **R** | **R** | S | **R** | S | **R** | S | **M** | S | S | **R** | 24 | >256 | 12 | U | U | U | U |
| C8:2:4 | **R** | **R** | **M** | **R** | S | **R** | S | **R** | S | S | **R** | 12 | >256 | 12 | U | U | U | U |
| C8:2:5 | **R** | **R** | **M** | **R** | S | **R** | S | **R** | S | S | **R** | 12 | >256 | 12 | U | U | U | U |
| C8:2:6 | **R** | **R** | **R** | **R** | S | **R** | S | **R** | S | S | **R** | 32 | >256 | 16 | U | U | U | U |
| C8:7:2 | **R** | **R** | S | **R** | S | **R** | S | **R** | S | S | **R** | 12 | >256 | 24 | U | U | U | U |
| C8:7:3 | **R** | **R** | **M** | **R** | S | **R** | S | **R** | S | S | **R** | 12 | >256 | 8 | U | U | U | U |
| C8:7:5 | **R** | **R** | S | **R** | S | **R** | S | **R** | S | S | **R** | 24 | >256 | 12 | U | U | U | U |
| C8:7:6 | **R** | **R** | **M** | **R** | S | **R** | S | **R** | S | S | **R** | 16 | >256 | 32 | U | U | U | U |
| C11:3:1 | **R** | **R** | S | **R** | S | **R** | S | **R** | S | S | **R** | 8 | 48 | >32 | U | U | U | U |
| C11:3:2 | **R** | **R** | **M** | **R** | S | **R** | S | **R** | S | S | **R** | 8 | 192 | >32 | U | U | U | U |
| C11:3:3 | **R** | **R** | S | **R** | S | **R** | S | **R** | S | S | **R** | 6 | 24 | >32 | U | U | U | U |
| C11:3:4 | **R** | **R** | S | **R** | S | **R** | S | **R** | S | S | **M** | 8 | 128 | >32 | U | U | U | U |
| C11:3:5 | **R** | **R** | **M** | **R** | S | **R** | S | **R** | S | S | **R** | 4 | 12 | >32 | U | U | U | U |
| C11:3:6 | **R** | **R** | S | **R** | S | **R** | S | **R** | S | S | **R** | 6 | 32 | >32 | U | U | U | U |
| C11:4:3 | **M** | **R** | **M** | **R** | S | **R** | S | **R** | S | S | S | 8 | 64 | 32 | U | U | U | U |
| C11:7:2 | **R** | **R** | **M** | **R** | S | **R** | S | **R** | S | S | **R** | 8 | >256 | 32 | U | U | U | U |
| C11:10:1 | **R** | **R** | S | **M** | S | **R** | S | **M** | S | S | S | 4 | 12 | 8 | U | U | U | U |
| C11:10:2 | **R** | **R** | S | **M** | S | **R** | S | **R** | S | S | S | 4 | 24 | 12 | U | U | U | U |
| C11:10:4 | S | **R** | S | **R** | S | **R** | S | **R** | S | S | S | 4 | 24 | 8 | U | U | U | U |
| C11:10:5 | **M** | **R** | S | **R** | S | **R** | S | **R** | S | S | **M** | 2 | 6 | 12 | U | U | U | U |
| C12:2:2 | **R** | **R** | S | **R** | S | **R** | S | **R** | S | S | **M** | 16 | >256 | >32 | U | U | U | U |
| C12:2:3 | **R** | **R** | **M** | **R** | S | **R** | S | **R** | S | S | **R** | 24 | >256 | >32 | U | U | U | U |
| C12:3:1 | **M** | **R** | **M** | **R** | S | **R** | S | **R** | S | S | **R** | 12 | >256 | >32 | U | U | U | U |
| C12:3:2 | **M** | **R** | S | **R** | S | **R** | S | **R** | S | S | **R** | 16 | >256 | >32 | U | U | U | U |
| C12:3:4 | **R** | **R** | S | **R** | S | **R** | S | **R** | S | S | **R** | 8 | >256 | >32 | U | U | U | U |
| C12:3:6 | **R** | **R** | **R** | **R** | S | **R** | S | **R** | S | S | **R** | 24 | >256 | >32 | U | U | U | U |
| C12:8:5 | **M** | **R** | S | **R** | S | **R** | S | **R** | S | S | **R** | 12 | >256 | >32 | U | U | U | U |
| C14:6:1 | **R** | **R** | **M** | **R** | S | **R** | S | **R** | S | S | **R** | 16 | >256 | 32 | U | U | U | U |
| C14:6:5 | **R** | **R** | **M** | **R** | S | **R** | S | **R** | S | S | **R** | 8 | >256 | 32 | U | U | U | U |
| C14:7:4 | **R** | **R** | **M** | **R** | S | **R** | S | **R** | S | S | **R** | 24 | >256 | 16 | U | U | U | U |
| C14:10:1 | **R** | **R** | **M** | **R** | S | **R** | S | **R** | S | S | **M** | 6 | >256 | 32 | U | U | U | U |
| C14:10:4 | **R** | **R** | **M** | **R** | S | **R** | S | **R** | S | S | **R** | U | U | 12 | U | U | U | U |
| C15:4:6 | **M** | **R** | S | **R** | S | **R** | S | **R** | S | S | **R** | U | U | >32 | U | U | U | U |
| C15:6:2 | **R** | **R** | **M** | **R** | S | **R** | S | **R** | S | S | S | U | U | U | U | U | U | U |
| C15:6:3 | **R** | **R** | **M** | **R** | S | **R** | S | **R** | S | S | **R** | >256 | >256 | U | U | U | U | U |
| C15:6:4 | **M** | **R** | S | **R** | S | **R** | S | **M** | S | S | S | 8 | 64 | U | U | U | U | U |
| C15:6:5 | **R** | **R** | **R** | **R** | S | **R** | S | **R** | S | S | **R** | >256 | >256 | U | U | U | U | U |
| C16:3:2 | **R** | **R** | **M** | **R** | S | **R** | S | **R** | S | S | **R** | 48 | >256 | U | U | U | U | U |
| C16:3:6 | **R** | **R** | **M** | **R** | S | **R** | S | **R** | S | S | **M** | >256 | >256 | U | U | U | U | U |
| C16:4:1 | **M** | **R** | **M** | **R** | S | **R** | S | **R** | S | S | **R** | 12 | >256 | U | U | U | U | U |
| C16:7:2 | **R** | **R** | **M** | **R** | S | **R** | S | **R** | S | S | **M** | >256 | >256 | U | U | U | U | U |
| C16:7:3 | **M** | **R** | S | **R** | S | **R** | S | **R** | S | S | **M** | >256 | >256 | U | U | U | U | U |
| C16:7:4 | **R** | **R** | **M** | **R** | S | **R** | S | **R** | S | S | **M** | >256 | >256 | U | U | U | U | U |
| C16:7:5 | **R** | **R** | **R** | **R** | S | **R** | S | **R** | S | S | **R** | >256 | >256 | U | U | U | U | U |
| C16:7:6 | S | **R** | **M** | **R** | S | **R** | S | **M** | S | S | **R** | >256 | >256 | U | U | U | U | U |
| C16:8:3 | S | **R** | S | **R** | S | **R** | S | **M** | S | S | **R** | 12 | >256 | U | U | U | U | U |
| C16:8:5 | S | **R** | S | **R** | S | **R** | S | **M** | S | S | **R** | 12 | >256 | U | U | U | U | U |
| C16:8:6 | S | **R** | S | **M** | S | **R** | S | **M** | S | S | S | 8 | >256 | U | U | U | U | U |
| C16:9:1 | **R** | **R** | S | **R** | S | **R** | S | **R** | S | S | **R** | 16 | >256 | U | U | U | U | U |
| C16:9:2 | **M** | **R** | S | **R** | S | **R** | S | **R** | S | S | **M** | 8 | >256 | U | U | U | U | U |
| C16:10:2 | **R** | **R** | S | **R** | S | **R** | S | **R** | S | S | **R** | >256 | >256 | U | U | U | >256 | U |
| C16:10:4 | S | **R** | S | **R** | S | **R** | S | **R** | S | S | **R** | 12 | >256 | U | U | U | U | U |
| C16:10:5 | **R** | **R** | S | **R** | S | **R** | S | **R** | S | S | **M** | 12 | >256 | U | U | U | U | U |
| C16:10:6 | **R** | **R** | **M** | **R** | S | **R** | S | **M** | S | S | **R** | >256 | >256 | U | U | U | U | U |
| C17:8:6 | **R** | **R** | **M** | **R** | S | **R** | S | **R** | S | S | **R** | >256 | >256 | U | U | U | U | U |
| C17:9:1 | S | **R** | S | **R** | S | **R** | S | **M** | S | S | **R** | 6 | >256 | U | U | U | U | U |
| C17:9:6 | S | **R** | S | **R** | S | **R** | S | **M** | S | S | **R** | 24 | >256 | U | U | U | U | U |
| C17:10:3 | **R** | **R** | **M** | **R** | S | **R** | S | **R** | S | S | **R** | 96 | >256 | U | U | U | U | U |
| C18:1:1 | S | **R** | S | **M** | S | **R** | S | **M** | S | S | **R** | 4 | >256 | U | U | U | U | U |
| C18:3:3 | S | **R** | S | **R** | S | **R** | S | **M** | S | S | S | 8 | >256 | U | U | U | U | U |
| C18:5:1 | **M** | **R** | S | **R** | S | **R** | S | **R** | S | S | **R** | 12 | >256 | U | U | U | U | U |
| C18:5:2 | S | **R** | S | **M** | S | **R** | S | **M** | S | S | S | 2 | 48 | U | U | U | U | U |
| C18:5:3 | **R** | **R** | S | **R** | S | **R** | S | **R** | S | S | **R** | >256 | >256 | U | U | U | U | U |
| C18:6:1 | **M** | **R** | S | **M** | S | **R** | S | **R** | S | S | **R** | 8 | >256 | U | U | U | U | U |
| C18:7:1 | **R** | **R** | **M** | **R** | S | **R** | S | **R** | S | S | **R** | >256 | >256 | U | U | U | U | U |
| C21:8:2 | **R** | **R** | **R** | **R** | S | **R** | S | **R** | S | S | **R** | 24 | >256 | U | U | U | U | U |
| C22:1:1 | S | **R** | S | **R** | S | **R** | S | **R** | S | S | S | >256 | >256 | U | U | U | U | U |
| C23:2:1 | **M** | **R** | S | **R** | S | **R** | S | **R** | S | S | **R** | 6 | >256 | U | U | U | U | U |
| C23:2:2 | **R** | **R** | S | **R** | S | **R** | S | **M** | S | S | **R** | 4 | >256 | U | U | U | U | U |
| C23:2:4 | **M** | **M** | S | **R** | S | **R** | S | **M** | S | S | **R** | U | U | U | U | U | U | U |
| C23:4:2 | S | **M** | S | **M** | S | **R** | S | **M** | S | S | **R** | U | U | U | U | U | U | U |
| Isolates = 148 | 31 | 139 | 67 | 147 |  | 148 |  | 148 |  |  | 102 |  |  |  |  |  |  |  |

AmC= Amoxicillin, Am10= Ampicillin, Cb100= Carbenicillin, CIP5= Ciprofloxacin, E15= Erythromycin, GM10= Gentamicin, D30= Doxycycline, VA30= Vancomycin, NA30= Nalidixic Acid, RA5= Rifampin, S10= Streptomycin

S= Susceptible, I= Increased Sensitivity, M= Moderate, R= Resistant, U= Untested
